# The variant senescence‐associated secretory phenotype induced by centrosome amplification constitutes a pathway that activates hypoxia‐inducible factor‐1α

**DOI:** 10.1111/acel.13766

**Published:** 2023-01-20

**Authors:** Selwin K. Wu, Juliana Ariffin, Shu Chian Tay, Remigio Picone

**Affiliations:** ^1^ Department of Cell Biology Harvard Medical School Massachusetts Boston USA; ^2^ Department of Pediatric Oncology Dana‐Farber Cancer Institute Massachusetts Boston USA; ^3^ Department of Surgery Cancer Research Institute, Beth Israel Deaconess Medical Center, Harvard Medical School Massachusetts Boston USA; ^4^ Mechanobiology Institute National University of Singapore Singapore; ^5^ Present address: Mechanobiology Institute & Department of Biological Sciences National University of Singapore Singapore

**Keywords:** ANGPTL4, centrosome, GEF Trio, HIF‐1α, microtubule organising center, paracrine invasion, Rac, reactive oxygen species, sasp, senescence

## Abstract

The senescence‐associated secretory phenotype (SASP) can promote paracrine invasion while suppressing tumour growth, thus generating complex phenotypic outcomes. Likewise, centrosome amplification can induce proliferation arrest yet also facilitate tumour invasion. However, the eventual fate of cells with centrosome amplification remains elusive. Here, we report that centrosome amplification induces a variant of SASP, which constitutes a pathway activating paracrine invasion. The centrosome amplification‐induced SASP is non‐canonical as it lacks the archetypal detectable DNA damage and prominent NF‐κB activation, but involves Rac activation and production of reactive oxygen species. Consequently, it induces hypoxia‐inducible factor 1α and associated genes, including pro‐migratory factors such as ANGPTL4. Of note, cellular senescence can either induce tumourigenesis through paracrine signalling or conversely suppress tumourigenesis through p53 induction. By analogy, centrosome amplification‐induced SASP may therefore be one reason why extra centrosomes promote malignancy in some experimental models but are neutral in others.

## Graphical abstract

We report that centrosome amplification induces a variant of SASP, which constitutes a pathway activating paracrine invasion. The centrosome amplification‐induced SASP is non‐canonical as it lacks the archetypal detectable DNA damage and prominent NF‐κB activation, but involves Rac activation by Trio and production of reactive oxygen species. Consequently, it induces hypoxia‐inducible factor 1α and associated genes, including pro‐migratory factors such as ANGPTL4. Of note, cellular senescence can either induce tumourigenesis through paracrine signalling or conversely suppress tumourigenesis through p53 induction. By analogy, centrosome amplification‐induced SASP may therefore be one reason why extra centrosomes promote malignancy in some experimental models but are neutral in others.

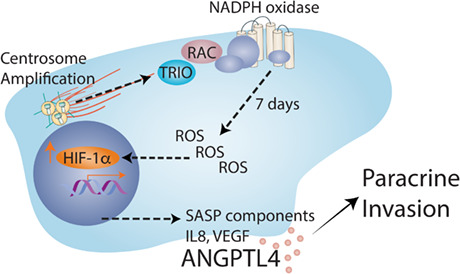

## 1 INTRODUCTION

Cellular senescence is characterised by several factors, including irreversible cell cycle arrest (Kuilman et al., 2010). In the context of cancer, it is an anti‐proliferative strategy cells adopt when responding to the activation of an oncogene. By suspending the division of cells at risk of malignant transformations and enabling the immune clearance of such cells, it prohibitively interferes with tumourigenesis (Perez‐Mancera et al., 2014).

Indeed, the senescence programme is much more complex than just the cessation of cell division, summoning various dynamic pathways and variable phenotypes (Schmitt, 2016). Importantly, senescent cells acquire the senescence‐associated secretory phenotype (SASP) (Pan et al., 2020), a prominent hallmark of senescence, and conventionally a product of persistent DNA damage and consequential regulation by NF‐κB (Kang et al., 2015; Chien et al., 2011). The SASP possesses a secretome containing paracrine factors, through which it communicates with and remodels its immediate microenvironment. This, intriguingly, leads to pathologically conflicting outcomes, concurrently fostering and inhibiting different aspects of tumour development (Coppe et al., 2010).

Centrosome amplification is one of the known triggers of cell cycle arrest (Ganem et al., 2014; Fava et al., 2017; Holland et al., 2012a). However, whether SASP is induced along with centrosome amplification‐induced proliferation arrest remains elusive. Centrosomes coordinate the assembly of microtubules during the spindle formation of cell division. A cell in the G1 phase typically has one centrosome, which is duplicated into two for mitosis. Thus, centrosome amplification can be deleterious to untransformed cells and activates signalling pathways which promote p53 stabilisation and cell proliferation arrest (Ganem et al., 2014). Strikingly, the deletion or suppression of p53 promotes spontaneous tumourigenesis in mice models (Sercin et al., 2016; Levine et al., 2017).

Though their comparability hints at a relation, the link between centrosome amplification and SASP is elusive. The senescence programme is variably characterised by several non‐exclusive markers, including constitutive DNA damage response signalling, senescence‐associated β‐galactosidase (SA‐βgal) activity, increased expression of the cyclin‐dependent kinase (CDK) inhibitor p21CIP1 (CDKN1A) and increased secretion of many extracellular factors through the SASP program (Sharpless & Sherr, 2015). Although many senescence‐associated markers result from alterations in transcription, the senescent phenotype is variable, attributing to difference in tissue‐type, cell‐type and senescence trigger (Sharpless & Sherr, 2015), and the dynamic nature of gene expression in senescent cells (Schmitt, 2016; Hoare et al., 2016; Lee & Schmitt, 2019). Despite the variabilities, a conserved transcriptome signature can be utilised as a useful marker to consistently identify senescent cells (Hernandez‐Segura et al., 2017). Here, we report a pathway for the centrosome amplification‐induced SASP variant to activate the hypoxia‐inducible factor 1α (HIF‐1α) in epithelial cells.

## 2 RESULTS

### 2.1 Centrosome amplification induced a variant senescence‐associated secretory phenotype independent of DNA damage

Supernumerary centrosomes were generated in cells by overexpressing Polo‐like kinase 4 (PLK4), the master regulatory kinase for centrosome duplication (Bettencourt‐Dias et al., 2005; Habedanck et al., 2005). PLK4 was transiently induced, and subsequent analysis was performed at time points where increased PLK4 mRNA expression was no longer detectable (Godinho et al., 2014). The majority of MCF10A cells (~80%) contained extra centrosomes 48 h after PLK4 induction (Figure S1a). In most experiments described below, cells with the transient overexpression of a truncated PLK4 mutant (Control 608) with kinase activity, which did not localise to the centrosomes (Guderian et al., 2010), were used as the negative control.

**FIGURE 1:**
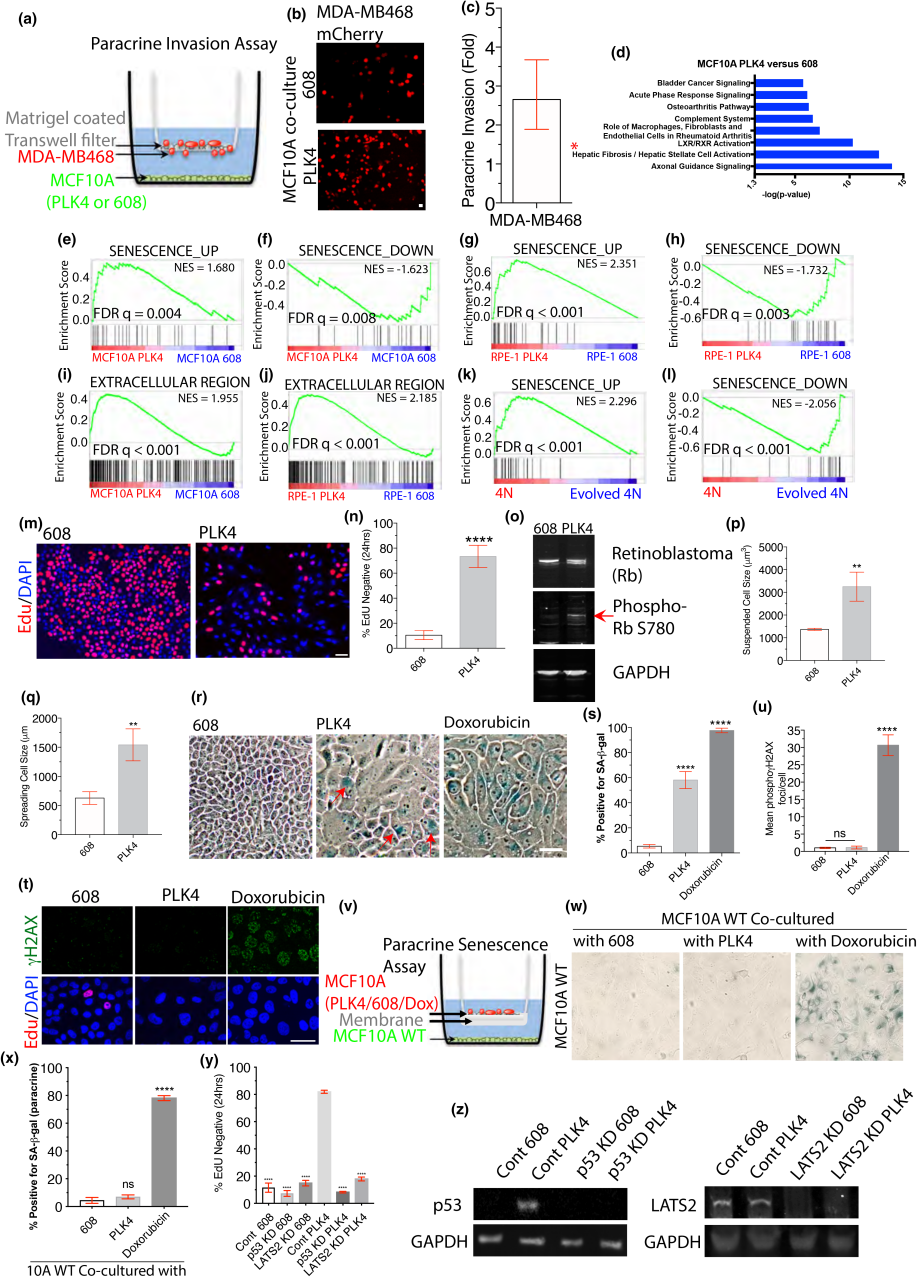
Centrosome amplification triggers a variant senescence‐associated secretory phenotype (SASP). (a) Experimental scheme for the matrigel‐coated transwell invasion experiment. Transwell inserts physically separate mCherry MDA‐MB468 cells (top chamber) from the indicated MCF10A cells (bottom chamber). Paracrine invasion is scored from the number of mCherry MDA‐MB468 cells that migrate to the bottom chamber. (b, c) MCF10A cells with centrosome amplification promote the invasion of MDA‐MB468 mCherry cells. Representative images (b) and fold increase (c) in mCherry MDA‐MB468 cells that crossed the matrigel‐coated transwell upon co‐culturing with the indicated MCF10A cells. (d) Centrosome amplification alters expression of genes related to cell motility and senescence. Ingenuity Pathway Analysis (IPA) of gene expression changes in MCF10A cells with centrosome amplification relative to controls revealing the top pathways altered by centrosome amplification. *Hepatic Fibrosis/ Hepatic Stellate Cell Activation is a senescence‐regulated process (Krizhanovsky et al., 2008). (e–h) Induction of senescence‐related gene expression in cells with centrosome amplification. Gene set enrichment analysis (GSEA) revealed strong enrichment of genes upregulated (e, g) or downregulated (f, h) with senescence in MCF10A cells with centrosome amplification (e, f) and RPE‐1 cells with centrosome amplification (g, h) relative to controls. NES: normalised enrichment score; FDR: false discovery rate. (i, j) Induction of secreted protein expression in cells with centrosome amplification. Gene set enrichment analysis (GSEA) revealed enrichment of genes annotated to the extracellular region in MCF10A cells with centrosome amplification (i) or RPE‐1 cells with centrosome amplification (j). NES: normalised enrichment score; FDR: false discovery rate. (k, l) Induction of senescence‐related gene expression in cells with centrosome amplification. Gene set enrichment analysis (GSEA) revealed strong enrichment of genes upregulated (k) or downregulated (l) with senescence in RPE‐1 tetraploids relative to “evolved tetraploids.” NES: normalised enrichment score; FDR: false discovery rate. (m, n) Proliferation arrest after centrosome amplification. Representative images (m) and quantification (n) of cells that cycled through S‐phase (24 hr. EdU‐label, red) and DAPI (blue) in MCF10A cells with (PLK4) and without (608) centrosome amplification. (o) Centrosome amplification induces retinoblastoma protein phosphorylation. Rb, Phospho Rb S780 and GAPDH (loading control) immunoblots of lysates from MCF10A cells with and without centrosome amplification. (p, q) Centrosome amplification increases cell size. Suspended (trypsinised) (p) and adherent (q) cell size measured for MCF10A cells with or without centrosome amplification. (r, s) Increased SA‐β‐Gal staining in cells with centrosome amplification. Representative images (r) and quantification (s) of SA‐β‐Gal (blue) staining of MCF10A cells with and without centrosome amplification or positive control with doxorubicin treatment. (t, u) Centrosome amplification does not induce DNA damage. Representative images (t) and quantification (u) of *γ*H2AX foci (green) in MCF10A cells with (PLK4) and without (608) centrosome amplification as compared to DNA damage from doxorubicin treatment. Cells in S‐phase are labelled with Edu (red) pulse and excluded from the quantification. (v, w, x) Centrosome amplification does not induce paracrine invasion. (v) Experimental scheme for the paracrine senescence assay. Transwell inserts ensure physical separation between MCF10A PLK4(top), 608(top) or wild‐type cells (bottom). Representative images (w) and quantification (x) of SA‐β‐Gal (blue) staining of MCF10A wild‐type cells that were co‐cultured with PLK4 or 608 or doxorubicin treated cells. (y, z) RNAi‐mediated knockdown of p53 or LATS2 releases MCF10A cells with centrosome amplification from proliferation arrest. (y) Quantification of control, p53 knockdown and LATS2 knockdown MCF10A cells with (PLK4) and without (608) centrosome amplification, which cycled through S‐phase (24 hr EdU‐label, red). (z) p53, LATS2 and GAPDH (loading control) immunoblots of lysates from control 608, p53 KD 608, LATS2 KD 608, control PLK4, p53 KD PLK4 and LATS2 KD PLK4 cells. Scale bar, 50 μm. All data are means ± SEM from *n* = 3 independent experiments, **p < 0.01, ****p < 0.0001; analysed with Student's t test except m, which was with one‐way ANOVA, Tukey's multiple comparison test. Scale bars, 50 μm.

SASP often induces paracrine invasion (Coppe et al., 2010). To determine whether cells with centrosome amplification could induce paracrine invasion, we used an established invasion assay with a matrigel‐coated transwell (Pan et al., 2020). MCF10A cells with and without supernumerary centrosomes were introduced into the bottom chamber of transwells, separated from the top chamber by a matrigel insert. The invasion of MDA‐MB468 cancer cells across the matrigel and filter barrier (Figure 1a) was then measured. Strikingly, cells with centrosome amplification generated a ~ 2.5‐fold increase in invading MDA‐MB468 cancer cells relative to the control (Figure 1a–c). Thus, soluble factors from cells with centrosome amplification can promote the cell motility of nearby invasive cells.

Next, we used RNA sequencing to identify the SASP factors released due to centrosome amplification. Transient PLK4 induction was used to trigger centrosome amplification in MCF10A cells and RPE‐1 cells, resulting in ~86% and ~ 91% of cells, respectively, having supernumerary chromosomes (Figure S1a, b). The transcriptomes of cells with (PLK4) and without (Control 608) centrosome amplification were compared 7 days post‐induction. For MCF10A cells, among the 1987 genes with significantly altered expression (fold change (FC) ≥ ±2, *q*‐value < 0.05), 1285 were upregulated after centrosome amplification, whereas for RPE‐1 cells, 49 genes were significantly upregulated in cells with centrosome amplification. In both cell types after centrosome amplification, Ingenuity Pathway Analysis (IPA) revealed that the differentially expressed genes (fold change ≥ ± 2, *q*‐value < 0.05) were enriched for terms associated with inflammation and the senescence‐associated secretory phenotype (SASP) (Krizhanovsky et al., 2008; Jeon et al., 2017; Childs et al., 2016) (Figure 1d and Figure S1c).

Subsequently, we queried the transcriptomes of cells with (PLK4) and without (Control 608) centrosome amplification for gene expression signatures of senescence and upregulation of secreted protein. Despite variabilities within the senescent phenotype, the transcriptome signatures have been able to consistently be used to identify senescent cells (Hernandez‐Segura et al., 2017). Thus, we employed this core senescent gene set to verify the manifestation of senescence in the whole‐transcriptome datasets analysed. Gene set enrichment analysis (GSEA) (Subramanian et al., 2005) in both MCF10A and RPE‐1 cells revealed a strong association between centrosome amplification and senescence (FDR *q* = 0.004, Figure 1e, FDR *q* = 0.008, Figure 1f, FDR *q* < 0.001, Figure 1g, FDR *q* = 0.003, Figure 1h), and secretion of extracellular region proteins (FDR *q* < 0.001, Figure 1i, FDR *q* < 0.001, Figure 1j). The top 50 genes in the “extracellular region” GSEA leading‐edge subset from both cell types contained a wide variety of secreted factors, such as extracellular matrix proteins, cytokines, angiogenic and pro‐invasive factors, including the SASP factor IL8 (Figure S1d, e).

As an orthogonal approach to examine the effects of centrosome amplification, we employed a PLK4 expression‐independent approach to generate extra centrosomes. We compared the transcriptomes of newly generated tetraploid cells from dicytochalasin B‐induced cytokinesis failure with doubled centrosome content to those of the control cells. Both parental diploid cells and “evolved” tetraploid cells that had spontaneously lost their extra centrosomes were used as controls (Ganem et al., 2014; Ganem et al., 2009). By IPA or GSEA with both data sets, the upregulated genes in the newly generated tetraploids were enriched for secreted extracellular region proteins (FDR q < 0.001, Figure S1f, g), and inflammatory and senescence‐associated pathway terms (FDR q < 0.001, Figure 1k, l and Figure S1h–k). The senescence‐associated pathway terms were similar to what we had observed in MCF10A cells exposed to 12Gy of γ‐irradiation (Figure S1l). Our data showed that the loss of extra centrosomes in tetraploids was sufficient to downregulate senescence‐related gene expression. Thus, the results from using orthogonal experimental approaches suggested that centrosome amplification induces senescence and cultivates a senescence‐associated secretome.

Senescence is a complex and multifaceted phenomenon. Under current conventions, cells are typically defined as senescent if they display proliferation arrest and at least two canonical features of senescence (Perez‐Mancera et al., 2014; Gorgoulis et al., 2019). In addition to proliferation arrest (Figure 1m, n) and SASP‐like gene expression patterns, centrosome amplification leads to the manifestation of other features of senescence. Consistent with the case of a p53‐dependent proliferation block, retinoblastoma phosphorylation was increased in cells with centrosome amplification (Figure 1o). Also consistent with the entry into senescence (Kuilman et al., 2010), centrosome amplification caused a ~ twofold increase in cell size as assayed by measuring the volume of cells in suspension following trypsinisation increase (Figure 1p) or the adherent cell area (~2.4‐fold increase, Figure 1q).

Stimulation of senescence‐associated beta‐galactosidase activity (~60% positive cells, Figure 1r, s) was also observed in cells with extra centrosomes, albeit at a lower intensity and frequency than was observed after extensive DNA damage. Persistent DNA damage response leading to DNA damage foci marked by *γ*H2AX, 53BP1 or ATM (Ataxia‐Telangiectasia Mutated) (Kang et al., 2015; Rodier et al., 2009) is required for a robust SASP. However, multiple studies have demonstrated that centrosome amplification does not cause detectable DNA damage in the nucleus (Ganem et al., 2014; Levine et al., 2017; Holland et al., 2012b). Strikingly, even ~8 days after the induction of extra centrosomes, when many features of senescence were apparent, we observed no significant increase in the percentage of cells positive for *γ*H2AX in the nucleus (Figure 1t, u). Therefore, we reasoned that the weaker senescence detected by senescence‐associated beta‐galactosidase activity was due to a lack of extensive and persistent DNA damage essential for a robust senescence phenotype. Consistent with a weaker senescence phenotype, we find that cells with centrosome amplification were unable to induce detectable paracrine senescence as well (Figure 1v–x), likely due to a lack of DNA damage signalling induction of robust inflammatory pathways. Nevertheless, cellular senescence depended on the centrosome amplification‐induced LATS2‐p53 pathway (Ganem et al., 2014; Holland et al., 2012a), as both LATS2 and p53 knockdown released the proliferation arrest due to centrosome amplification (Figure 1y, z and Figure S1m).

### 2.2 Centrosome amplification promotes the induction of ANGPTL4

**FIGURE 2:**
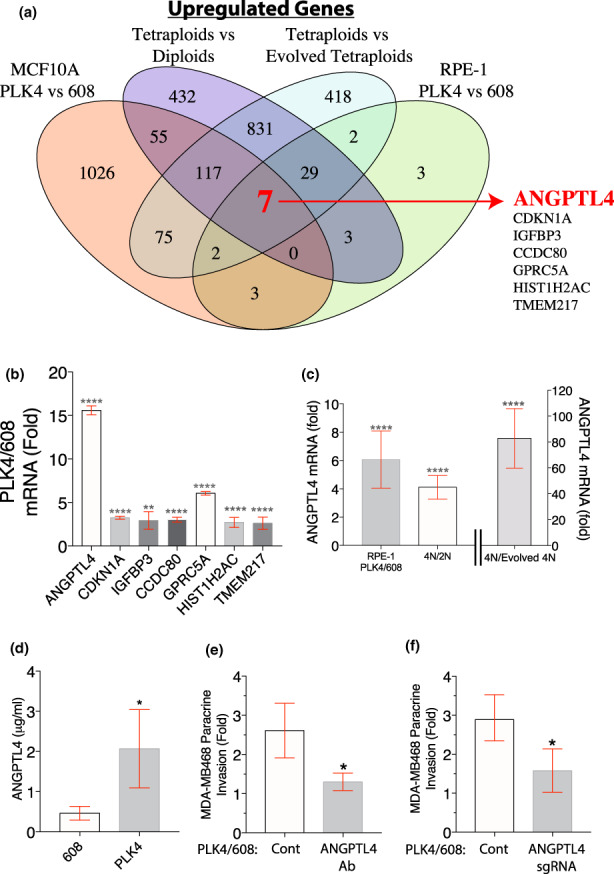
Centrosome amplification‐induced ANGPTL4. (a) Seven genes are upregulated by centrosome amplification from all experimental conditions, in two cell types. Venn diagram for the indicated comparisons showing the overlap of upregulated genes from RNA‐Seq (twofold change, *p* (adjusted) < 0.05). (b) Among the seven commonly upregulated genes, ANGPTL4 expression is the most strongly induced by centrosome amplification. Fold induction for this group of genes from RNA‐Seq of MCF10A cells with centrosome amplification relative to controls. (c) Induction of ANGPTL4 expression in cells with centrosome amplification. ANGPTL4 mRNA fold changes from RNA‐Seq in RPE‐1 cells in PLK4 versus 608, tetraploids versus parental diploids and tetraploids versus evolved tetraploids. (d) Induction of ANGPTL4 secretion in cells with centrosome amplification. ELISA analysis of secreted ANGPTL4 from cells with or without centrosome amplification. (e) Inhibition of ANGPTL4 compromises paracrine invasion stimulated by cells with centrosome amplification. Shown is the fold increase in MDA‐MB468 cells in the bottom chamber of the transwell in the presence of either control IgG or an ANGPTL4 blocking antibody, co‐cultured with the indicated MCF10A cells. (f) CRISPR‐mediated gene disruption of ANGPTL4 in cells with centrosome amplification inhibits induction of paracrine invasion. Fold increase in MDA‐MB468 cells that crossed the Matrigel‐coated transwell filter upon co‐cultured with control or sgANGPTL4 MCF10A cells with centrosome amplification. All data are means ± SEM from *n* = 3 independent experiments, *p < 0.05; analysed with Student's t test. **p < 0.01, ****p < 0.0001; adjusted *p*‐value (grey) analysed with DESeq2 Wald test.

Next, we sought to identify strongly and differentially expressed genes common to both RPE‐1 and MCF10A, with cytokinesis failure, in which centrosome amplification was induced. From our gene expression profiles among the different cell lines, seven genes were consistently upregulated under all experimental conditions (Figure 2a, Figure S2a and Table S1). These included the genes for the cyclin‐dependent kinase inhibitor p21 (Figure S2b), which was induced in all known examples of senescence, and IGFBP3, a known inducer of senescence (Kim et al., 2007) (Figure 2a, b). Among all the conditions and cell types, the expression of ANGPTL4 was the most prominent post‐centrosome amplification (up to ~15‐fold induction in MCF10A cells, p < 0.001 and approximately fivefold in RPE‐1 cells, Figure 2b, c). The induction of ANGPTL4 protein was confirmed using an enzyme‐linked immunosorbent assay (ELISA; Figure 2d). More importantly, inhibition of ANGPTL4 with a blocking antibody or by CRISPR‐mediated gene disruption significantly reduced paracrine migration stimulated by MCF10A cells with extra centrosomes (Figure 2e, f and Figure S2c–f). These findings are notable because ANGPTL4 is a well‐established pro‐invasive factor that activates Rac signalling and disrupts cell–cell contacts (Zhu et al., 2011; Huang et al., 2011; Padua et al., 2008; Zhang et al., 2012). As expected, the increased expression of ANGPTL4 is a common feature of SASP (Zhang et al., 2018).

### 2.3 Centrosome amplification activates HIF‐1α induction of ANGPTL4

**FIGURE 3:**
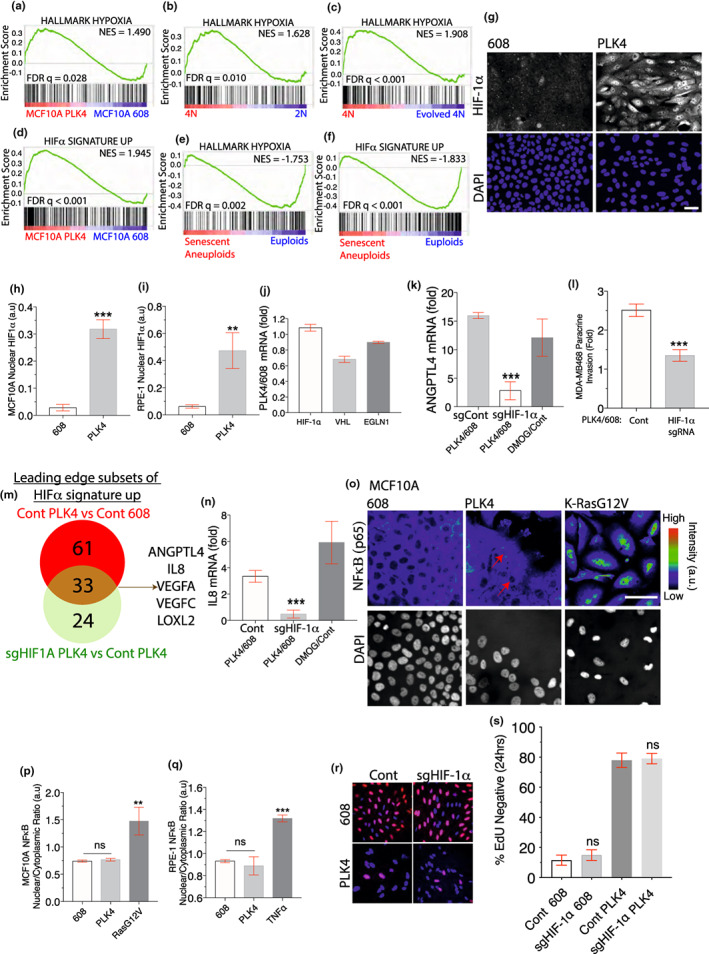
Centrosome amplification activates HIF‐1α. (a–c) Induction of the expression of hypoxia‐inducible genes in cells with centrosome amplification. GSEA revealed strong enrichment of an annotated hypoxia hallmark gene set in cells with centrosome amplification. RNA‐Seq data sets being compared are indicated at the bottom of the GSEA plots for MCF10A cells (a) and RPE‐1 cells (b and c). (d) A custom‐generated HIF‐1α signature up gene set in MCF10A cells is strongly induced by centrosome amplification in MCF10A cells. (e, f) Unlike centrosome amplification, aneuploidy‐induced senescence suppresses expression of the hallmark hypoxia (e) and custom‐generated MCF10A HIF‐1α signature up (f) gene sets. (g–i) Induction of nuclear HIF‐1α after centrosome amplification‐induced senescence. Representative images (g) and automated quantification of nuclear HIF‐1α in MCF10A cells (h) or RPE‐1 cells (i) with or without centrosome amplification (see online A. Appendix Methods and Figure S4). (j) Centrosome amplification does not alter the expression of HIF‐1α, VHL and EGLN1. Fold difference of HIF‐1α, VHL and EGLN1 expression levels from MCF10A cells RNA‐Seq data sets with or without centrosome amplification. (k) CRISPR‐mediated gene disruption of HIF‐1α reduces ANGPTL4 expression induced by centrosome amplification. The relative abundance of ANGPTL4 mRNA from RNA‐Seq in centrosome amplification or control hypoxia mimetic DMOG‐treated cells is expressed as the fold change with respect to control cells. Centrosome amplification and control 608 cells are subjected to CRISPR‐mediated gene disruption of HIF‐1α and compared with CRISPR non‐targeting sgRNA controls. (l) CRISPR‐mediated gene disruption of HIF‐1α in cells with centrosome amplification inhibits the induction of paracrine invasion. Fold increase in MDA‐MB468 cells that invaded across the Matrigel‐coated transwell when co‐cultured with control or sgHIF‐1α MCF10A cells with centrosome amplification. (m) Forty‐nine genes are significantly induced by centrosome amplification‐induced HIF‐1α activation. Venn diagram for the indicated comparisons showing the overlap of the leading‐edge subsets of genes from GSEA of HIF‐1α‐induced genes. (n) CRISPR‐mediated gene disruption of HIF‐1α reduces IL8 expression induced by centrosome amplification. Relative abundance of IL8 mRNA from RNA‐Seq in centrosome amplification or DMOG‐treated cells is expressed as the fold change relative to controls. Centrosome amplification and control cells are subjected to CRISPR‐mediated gene disruption of HIF‐1α and compared with the CRISPR non‐targeting control. (o) Lack of NF‐κB nuclear accumulation after centrosome amplification. Representative images of p65 RELA (NF‐κB) and DAPI (grey) in MCF10A cells with and without centrosome amplification as compared with a K‐RasG12V positive control. (p, q) Quantification of p65 RELA (NF‐κB) nuclear/cytoplasmic ratio in MCF10‐A (p) and RPE‐1 (q) cells with or without centrosome amplification as compared to K‐RasG12V overexpression (p) or TNFα treatment (q). (r, s) HIF‐1α depletion did not release cells with centrosome amplification from proliferation arrest. Representative images (r) and quantification (s) of control and sgHIF‐1α cells with (PLK4) and without (608) centrosome amplification that cycled through S‐phase (24 hr. EdU‐label, red) in MCF10A cells. All data are means ± SEM from *n* = 3 independent experiments, **p < 0.01, ***p < 0.001, ****p < 0.0001; analysed with one‐way ANOVA, Tukey's multiple comparison test. Scale bars, 50 μm.

We next addressed the mechanism by which centrosome amplification induces the expression of ANGPTL4. ANGPTL4 is an established target of HIF‐1α, the master regulator of the hypoxic response (Zhang et al., 2012; Li et al., 2011; Semenza, 2012). Thus, we considered the possibility that HIF‐1α could be activated in a centrosome amplification‐induced SASP. HIF‐1α are crucial transcription factors that regulate oxygen sensing and mediate the hypoxic response (Kietzmann et al., 2016). Activation of HIF‐1α triggers the transcription of genes involved in cancer (Miroshnikova et al., 2016) and immunity (Palazon et al., 2014), including cytokines IL8, IL6, ANGPTL4, VEGF and PDGF, which are commonly observed in SASP (Coppe et al., 2010; Zhang et al., 2012; Maxwell et al., 2007; Liu et al., 2009; Yoshida et al., 2006). Consistent with this idea, GSEA demonstrated that a hallmark hypoxia gene signature is present in both MCF10A and RPE‐1 cells with PLK4‐induced centrosome amplification (FDR *q* = 0.028, Figure 3a and FDR *q* < 0.001, Figure S2g). Additionally, newly generated tetraploid RPE‐1 cells with extra centrosomes also exhibited an enriched hypoxia signature as compared to parental diploid cells (FDR *q* = 0.010, Figure 3b) or evolved tetraploids lacking extra centrosomes (FDR *q* < 0.001, Figure 3c).

To rigorously define a relevant hypoxia gene set in MCF10A cells, we derived a gene expression signature of HIF‐1α activation from transcriptomic analysis of MCF10A cells treated with Dimethyloxalylglycine, N‐(Methoxyoxoacetyl)‐glycine methyl ester (DMOG). DMOG promotes the steady‐state accumulation of HIF‐1α by inhibiting EGLN/PHD prolyl hydroxlases (Kaelin Jr., 2005). Strikingly, this HIF‐1α activation gene set was significantly enriched in both MCF10A and RPE‐1 cells after centrosome amplification (FDR *q* < 0.001, Figure 3d and FDR *q* = 0.048, Figure S2h). By contrast, a previously reported data set for aneuploidy‐induced senescence in RPE‐1 cells (Santaguida et al., 2017) indicated that both the hallmark hypoxia gene set (FDR *q* = 0.002, Figure 3e) and our DMOG‐induced gene set (FDR *q* < 0.001, Figure 3f) were suppressed in aneuploid cells, underscoring the differences between the effects of centrosome amplification and aneuploidy (Godinho et al., 2014).

Consistent with these gene expression results, using a HIF‐1α‐specific antibody (Figure S2i–k), we found that cells with extra centrosomes exhibited a marked increase in nuclear HIF‐1α (Figure 3g–i Figure S2l,m). The HIF‐1α accumulation was post‐transcriptional, because centrosome amplification did not affect the HIF‐1α mRNA levels (Figure 3j). Moreover, centrosome amplification did not affect the expression of proteins involved in HIF‐1α destruction (Figure 3j). More importantly, CRISPR‐mediated gene disruption of HIF‐1α in cells with centrosome amplification blocked the activation of HIF‐1α‐regulated genes (FDR *q* = 0.014, Figure S2n, o), attenuated the induction of ANGPTL4 (Figure 3k) and reduced the capacity of these cells to promote paracrine migration (Figure 3l and Figure S2p). A total of 33 genes induced by centrosome amplification were critically dependent on HIF‐1α, including well‐established HIF‐1α targets such as VEGFA, VEGFC, ANGPTL4, LOXL2 and IL8 (Figure 3m and Table S1). Among these, IL8 is an important cytokine that is known to be regulated by HIF‐1α (Kim et al., 2006), as such, HIF‐1α disruption in cells with centrosome amplification reduced IL8 induction (Figure 3n).

The induction of HIF‐1α by centrosome amplification occurred regardless of whether cells were cultured under ambient oxygen tension (21% O_2_) or conditions of physiological normoxia/atmospheric hypoxia (5% O_2_). Gene expression profiles of RPE‐1 cells with or without centrosome amplification were obtained after ~7 days in culture in 5% O_2_. Consistent with our previous results obtained using ambient oxygen concentrations, cells in 5% oxygen with centrosome amplification exhibited strong upregulation of gene sets annotated for “extracellular proteins” (FDR *q* < 0.001, Figure S3a), “senescence” (FDR *q* < 0.001, FDR *q* = 0.035, Figure S3b, c) “hypoxia” and “HIF‐1α activation” (FDR *q* < 0.001, FDR *q* = 0.004, Figure S3d,e). Moreover, in physiological normoxia, we observed an enlarged adherent cell size, a ~ fivefold increase in nuclear HIF‐1α (Figure S3f, g), and triggering of ANGPTL4 and p21 expression after centrosome amplification (Figure S3h).

Notably, the activation of HIF‐1α that we have identified here is a non‐canonical contributor of the SASP secretome, since NF‐κB is typically thought to be the primary determinant of the SASP secretome (Kang et al., 2015), and DNA damage signalling had been shown to activate NF‐κB through the transcription factor GATA4(Kang et al., 2015; Rodier et al., 2009). However, centrosome amplification did not inflict pronounced DNA damage (Figure 1t, u). As a consequence, quantitative imaging indicated that centrosome amplification in three cell lines did not result in any detectable increase in the nuclear localisation of NF‐κB (Figure 3o–q and Figure S3i). Because the secreted factors regulated by NF‐κB reinforce senescence (Kang et al., 2015), the poor NF‐κB activation likely contributed to the weaker induction of senescence‐associated beta‐galactosidase activity by centrosome amplification (Figure 1r,s). Thus, centrosome amplification induced a variant SASP that lacked NF‐κB activation, although evidence suggested that hypoxia and HIF‐1α activation can prevent or delay cells from entering senescence (Welford & Giaccia, 2011). However, HIF‐1α activation only associates or accompanies cellular senescence but not directly inducing senescence, as depletion of HIF‐1α was insufficient to release cells from proliferation arrest (Figure 3r, s). Nevertheless, the HIF‐1α activity of cells after entering senescence has not been studied. Our data show that centrosome amplification‐induced SASP constitutes the activation of HIF‐1α.

### 2.4 Centrosome amplification activates HIF‐1α through induction of ROS

**FIGURE 4:**
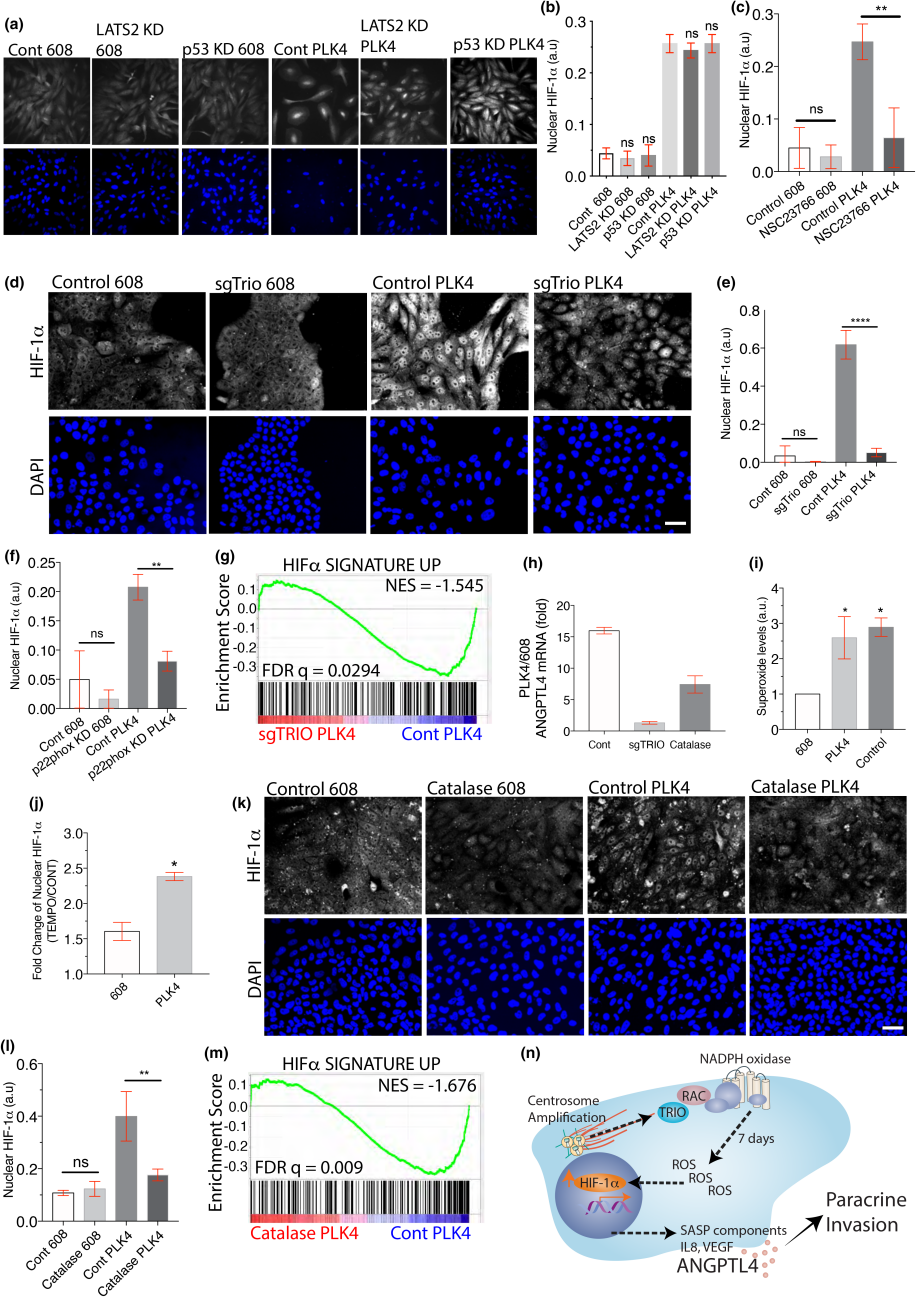
Centrosome amplification‐induced ROS. (a, b) RNAi‐mediated knockdown of p53 or LATS2 does not affect nuclear HIF‐1α accumulation after centrosome amplification. Shown are representative images (a) and quantification (b) of nuclear HIF‐1α levels in the indicated cells. (c) Small molecule Rac‐1 inhibition prevents the accumulation of nuclear HIF‐1α after centrosome amplification. The indicated MCF10A cells were treated with 50 μm NSC23766 or vehicle and HIF‐1α nuclear accumulation was measured. (d, e) CRISPR‐mediated gene disruption of TRIO blocks nuclear HIF‐1α accumulation after centrosome amplification. Gene targeting of a pool of cells was performed in the indicated MCF10A cells prior to the initiation of centrosome amplification. Shown are representative image (d) and quantification (e) of nuclear HIF‐1α levels in the indicated cells. (f) siRNA knockdown of p22^phox^ inhibits nuclear HIF‐1α accumulation after centrosome amplification. (g) Trio is required for the upregulation of HIF‐1α‐induced genes in MCF10A cells with centrosome amplification. Shown is a GSEA plot comparing cells with centrosome amplification with or without TRIO gene disruption. (h) The induction of ANGPTL4 by centrosome amplification requires Trio and ROS. Shown is the fold induction of ANGPTL4 from RNA‐Seq after centrosome amplification in MCF10A cells after the indicated treatments. (i) Accumulation of superoxide after centrosome amplification. Superoxide levels were measured by dihydroethidium labelling in MCF10A cells with and without centrosome amplification. Pyocyanin treatment is the positive control. (j) Conversion of superoxide into hydrogen peroxide further induces nuclear HIF‐1α accumulation in cells with centrosome amplification. Shown are the fold changes in nuclear HIF‐1α in the indicated MCF10A cells after treatment with TEMPOL. (k, l) Catalase blocks the nuclear accumulation of HIF‐1α in cells with centrosome amplification. Representative images (k) and quantification (l) of HIF‐1α in the indicated MCF10A cells with and without catalase medium addition. (m) GSEA showing that catalase treatment prevents the upregulation of the custom HIF‐1α signature up gene set in MCF10A cells. All data are means ± SEM from *n* = 3 independent experiments, *p < 0.05, **p < 0:01, ***p < 0:001, ****p < 0:0001; analysed with one‐way ANOVA, Tukey's multiple comparison test. Scale bars, 50 μm. (n) Model for centrosome amplification‐induced SASP.

Although LATS2 and p53 are responsible for centrosome amplification‐induced proliferation arrest (Ganem et al., 2014; Holland et al., 2012a) (Figure 1y, z, Figure S1m), LATS2 or p53 knockdown did not block the induction of HIF‐1α (Figure 4a, b). Thus, we turned our focus to reactive oxygen species (ROS). ROS production is induced by inflammatory cytokines, a common feature of senescence and an established trigger for HIF‐1α activation (Kaelin Jr., 2005; Chandel et al., 2000). Therefore, we hypothesised that increased levels of ROS could provide a link between centrosome amplification and HIF‐1α activation. Centrosome amplification can activate the Rac1 GTPase (Godinho et al., 2014). Among its many functions, Rac promotes ROS production because it is a stoichiometric subunit of NADPH oxidase (Zhu et al., 2011; Cheng et al., 2006). Therefore, we tested the hypothesis that post‐centrosome amplification, the Rac‐NADPH oxidase generates ROS to activate HIF‐1α. Consistent with this hypothesis, pharmacological inhibition of Rac (Figure 4c and Figure S4a), CRISPR‐mediated gene disruption of the Rac GTPase exchange factor, Trio (van Haren et al., 2014; Mizrahi et al., 2005; Muller et al., 2020) (Figure 4d, e and Figure S4b, c) and siRNA knockdown of a key component of the NADPH oxidase, p22^phox^ (Figure 4f and Figure S4d, e, f), all significantly reduced HIF‐1α nuclear accumulation in cells with centrosome amplification. Using RNA sequencing, we found that CRISPR‐mediated gene disruption of Trio also suppressed gene expression signatures associated with HIF‐1α activation, including the induction of ANGPTL4, without significantly affecting HIF‐1α regulated genes in control cells (Figure 4g (FDR *q* = 0.0294), h and, Figure S4g (FDR *q* = 0.044), S4h).

Centrosome amplification induces Rac activation (Ganem et al., 2014; Godinho et al., 2014). When Rac is activated, NADPH oxidase complex can generate superoxide (Abo et al., 1991). Using dihydroethidium labelling, we found that cells with centrosome amplification did indeed exhibit higher levels of superoxide (Figure 4i and Figure S5a). Cells convert superoxide into the more stable hydrogen peroxide by dismutation (Halliwell & Gutteridge, 2015) and both cellular superoxide and hydrogen peroxide promote HIF‐1α stabilisation (Chandel et al., 2000; Kaewpila et al., 2008). However, when we accelerated the conversion of superoxide into hydrogen peroxide by treating cells with TEMPOL (4‐hydroxy‐2,2,6,6‐tetramethyl piperidin‐1‐oxyl) (Kaewpila et al., 2008), nuclear HIF‐1α levels were further increased (Figure 4j and Figure S5b), suggesting that hydrogen peroxide is a major ROS conferring HIF‐1α stabilisation after centrosome amplification. Next, we added catalase to the culture medium to directly deplete hydrogen peroxide (Goyal et al., 2004). Strikingly, in cells with centrosome amplification, the catalase treatment prevented nuclear HIF‐1α accumulation and suppressed HIF‐1α‐associated gene expression, including the transcriptional induction of ANGPTL4 (Figure 4k–m (FDR *q* = 0.009), and Figure S5c). Thus, it is suggested that the Rac‐NADPH oxidase complex generated ROS, activated HIF‐1α in cells with extra centrosomes. Although ROS induction of HIF‐1α is a prominent feature of the SASP from centrosome amplification, elevated levels of ROS has been observed in many forms of senescence (Kuilman et al., 2010).

The hypoxia signalling may not be specific to centrosome amplification‐induced SASP. Consistent with previous evidence that gamma irradiation activates HIF‐1α (Moeller et al., 2004), nuclear HIF‐1α indeed accumulated in γ‐irradiation‐induced senescent cells, and we also observed the enrichment of hypoxia‐induced genes and a HIF‐1α activation signature (Figure S5d–g). HIF‐1α is also known to be activated by Ras signalling (Chan et al., 2002). Accordingly, we observed the enrichment of hypoxia‐induced genes and a HIF‐1α activation signature in published data sets (Acosta et al., 2013), whereby senescence was induced by oncogene activation (FDR *q* < 0.001, Figure S5h, i). Although the senescence phenotype exhibits significant variability across different cell lines and with different approaches to induce senescence (Hernandez‐Segura et al., 2017), our data showed that the expression of hypoxia‐induced genes could still be detected in other forms of senescence as well.

## 3 DISCUSSION

Here, we have demonstrated that centrosome amplification leads to the acquisition of a variant senescence‐associated secretory phenotype, which promotes paracrine invasion. The SASP has been well‐established to generate distinct phenotypic outcomes, promoting cancer invasion (Angelini et al., 2013; Kim et al., 2017) in many contexts, but notably, also suppressing tumourigenesis by inducing growth arrest (Santaguida et al., 2017; Eggert et al., 2016). Thus, drawing parallels from the aforementioned distinctive outcomes of conventional SASP has led us to speculate that centrosome amplification‐induced SASP is also a mechanism that stimulates tumour progression in some models (Levine et al., 2017; Godinho et al., 2014), but may suppress tumourigenesis in others due to the effects of growth suppression (Vitre et al., 2015; Kulukian et al., 2015). Consistent with our speculation, we found that cells with centrosome amplification have impaired proliferation but still promote paracrine invasion.

It is important to note that the induction of SASP does not, per se, depend on proliferation arrest (Kang et al., 2015; Rodier et al., 2009; Coppe et al., 2008; Coppe et al., 2011). Instead, mounting evidence indicates that although p53 induces proliferation arrest and the SASP share common initiating triggers, both phenomena are sustained by independent signalling pathways (Kang et al., 2015; Coppe et al., 2008). Thus, when p53 activation is compromised, cells may still proliferate but at the same time drive the secretory phenotype, resulting in paracrine invasion (Sercin et al., 2016; Coppe et al., 2008). Consistent with this hypothesis, deletion of p53 enables tumour formation in mice with centrosome amplification (Sercin et al., 2016; Levine et al., 2017).

The secretory phenotype we described has much in common with conventional SASP; nevertheless, there are some mechanistic differences between the two. Canonical SASP is induced by DNA damage and largely controlled by the transcription factor NF‐κB (Kang et al., 2015; Chien et al., 2011; Rodier et al., 2009). By contrast, centrosome amplification‐induced senescence lacks detectable DNA damage, thus is not accompanied by downstream nuclear translocation of p65/RelA/NF‐κB. The subtleness of the senescence phenotype induced by centrosome amplification than DNA damage made it more challenging to pinpoint, but it is still a significant source of SASP. Indeed, this secretory pathway has been studied at an earlier time window before full blown senescence (Schmitt, 2016; Arnandis et al., 2018), as a distinct secretory phenotype generated by extra centrosomes and was termed extra centrosomes associated secretory phenotype (ECASP) instead. It has been demonstrated that centrosome amplification facilitates the release of pro‐invasive factors such as IL‐8, ANGPTL4, and GDF‐15(Arnandis et al., 2018). Furthermore, it is recently reported that increased ROS levels in cells with extra centrosomes compromise lysosomal function and change the fate of mutivescular bodies towards fusing with the plasma membrane, resulting in the increased secretion of small extracellular vesicles (Adams et al., 2021). Thus, the activation of secretory trafficking mechanisms by centrosome amplification and ROS can further augment the upstream transcriptional activation of HIF‐1α.

There has been evidence suggesting that hypoxia and HIF‐1α activation can prevent or delay cells from entering senescence (Welford & Giaccia, 2011) through the expression of a HIF‐1α‐induced macrophage inhibitory factor. However, such expression had not been observed in our cells after centrosome amplification (data not shown). The activation of HIF‐1α by centrosome amplification is mediated by the increased production of ROS, as HIF‐1α activation is abrogated by perturbations to the NADPH oxidase or by catalase. Additionally, elevated levels of ROS, a canonical trigger for HIF‐1α activation, are observed in many forms of senescence. Consistent with elevated ROS, we detected expression of hypoxia‐induced genes in existing data sets, whereby senescence was induced by replicative exhaustion or oncogene activation. Moreover, we found that nuclear HIF‐1α accumulates in senescent cells after gamma irradiation. To our knowledge, the expression of hypoxia‐induced genes as a general feature of senescent cells has not been studied.

In summary, our findings demonstrated that centrosome amplification promotes a senescence‐associated secretory phenotype (SASP) that constitutes a pathway activating HIF‐1α (Figure 4 n). This variant senescence phenotype lacks detectable DNA damage and downstream NF‐κB activation. SASP has been well‐established to generate diverse phenotypic outcomes: promoting tumour invasion in many contexts (Angelini et al., 2013; Kim et al., 2017; Gluck et al., 2017; Dou et al., 2017), but also suppressing tumour growth through cellular proliferation arrest and immune response (Santaguida et al., 2017; Eggert et al., 2016). Importantly, if the tumour suppression mechanism of cellular senescence, such as the proliferation barrier, is not overcome, then instead of promoting tumourigenesis, the senescent cells would have a poor survival rate. Thus, the distinct consequences of SASP may be the reason why centrosome amplification promotes malignancy in some models (Levine et al., 2017; Godinho et al., 2014) but are neutral (Vitre et al., 2015; Kulukian et al., 2015) in others. Consistent with our speculation, centrosome amplification promotes spontaneous tumourigenesis in mice models only when the p53‐dependent proliferation block is either removed or becomes suppressed (Sercin et al., 2016; Levine et al., 2017).

## AUTHOR CONTRIBUTIONS

Selwin K. Wu conceived the project with inputs from Remigio Picone. Selwin K. Wu performed most of the experiments, except for ELISA and the MCF10A RNA‐Seq experiment in figure 1, which were performed and analysed by Remigio Picone. Remigio Picone made the original observation and analysis of centrosome amplification inducing paracrine motility through secreted factors including ANGPTL4. Selwin K. Wu and Shu Chian Tay wrote the manuscript. Selwin K. Wu analysed data. Juliana Ariffin and Shu Chian Tay edited the manuscript.

## CONFLICT OF INTEREST

The authors declare that the research was conducted in the absence of any commercial or financial relationships that could be construed as a potential conflict of interest.

## Supporting information


**Figure S1** Features of the centrosome amplification SASP. (a, b) Fraction of control (608) and PLK4‐induced MCF10A (a) or RPE‐1 (b) cells with more than two centrosomes was determined by immunostaining of centrin‐1. Representative images and quantification of MCF10A cells with (PLK4) and without (608) centrosome amplification. Scale bars, 5 μm. Data are means ± SEM from *n* = 2 independent experiments. (c) Centrosome amplification alters expression of genes related to cell motility and senescence. Ingenuity Pathway Analysis (IPA) of gene expression changes in RPE‐1 cells with centrosome amplification relative to controls revealing the top pathways altered by centrosome amplification. Hepatic Fibrosis/ Hepatic Stellate Cell Activation is a SASP‐regulated process (Krizhanovsky et al., 2008). (d, e) Induction of the expression of secreted proteins in cells with centrosome amplification. Heatmap showing the leading‐edge enrichment of the top 50 extracellular protein expression upregulated in MCF10A (d) and RPE‐1 (e) cells with centrosome amplification relative to control. (f, g) Induction of secreted protein expression in cells with centrosome amplification. Gene set enrichment analysis (GSEA) revealed enrichment of genes annotated to be the extracellular region in tetraploids relative to either parental diploids (f) or evolved tetraploids (g). NES: normalised enrichment score; FDR: false discovery rate. (h, i) Centrosome amplification alters expression of genes related to senescence. Ingenuity Pathway Analysis (IPA) of gene expression changes in tetraploids relative to either parental diploids (h) or evolved tetraploids (i) revealing the top pathways altered by centrosome amplification. *Hepatic Fibrosis/ Hepatic Stellate Cell Activation is a senescence‐regulated process (Krizhanovsky et al., 2008). (j, k) Heatmap showing the leading‐edge enrichment of the top 50 extracellular protein expression upregulated in tetraploids relative to either parental diploids (j) or evolved tetraploids (k). (l) γ‐irradiation alters expression of genes related to DNA damage‐induced senescence. Ingenuity Pathway Analysis (IPA) of gene expression changes in MCF10A cells exposed to 12Gy of γ‐irradiation relative to controls revealing the top pathways altered by DNA damage‐induced senescence. (m) siRNA knockdown of p53 and LATS2 release MCF10A cells with centrosome amplification from proliferation arrest. Representative images of control, p53 knockdown and LATS2 knockdown MCF10A cells with (PLK4) and without (608) centrosome amplification that cycled through S‐phase (24 hr. EdU‐label, red). Scale bar, 50 μm.
**Figure** **S2**. Gene expression altered by centrosome amplification, a functional requirement for ANGPTL4 (a) Zero genes are commonly downregulated by centrosome amplification from all experimental conditions. Venn diagram for the indicated comparisons showing the overlap of downregulated genes from RNA‐Seq (twofold change, *q* < 0.05). (b) Induction of p21 expression in cells with centrosome amplification. p21 mRNA fold changes from RNA‐Seq in RPE‐1 and MCF10A cells in PLK4 versus 608, tetraploids versus parental diploids and tetraploids versus evolved tetraploids. Data are means ± SEM from *n* = 3 independent experiments, ****p < 0:0001; adjusted *p*‐value (grey) analysed with DESeq2 Wald test. (c) CRISPR‐mediated gene disruption of ANGPTL4 does not affect the induction of extra centrosomes. The fraction of control (608) and PLK4 induced sgANGPTL4 MCF10A cells with more than two centrosomes, determined by immunostaining of centrin‐1. Data are means ± SEM from *n* = 2 independent experiments. (d) CRISPR‐mediated gene disruption of ANGPTL4. ANGPTL4 and GAPDH (loading control) immunoblots of lysates from doxycycline‐uninduced control PLK4, sgANGPTL4 PLK4, control 608 and sgANGPTL4 608 cells. (e) Inhibition of ANGPTL4 compromises paracrine invasion stimulated by cells with centrosome amplification. Shown is the representative image of MDA‐MB468 cells in the bottom chamber of the transwell in the presence of either control IgG or an ANGPTL4 blocking antibody, co‐cultured with the indicated MCF10A cells. (f) CRISPR‐mediated gene disruption of ANGPTL4 in cells with centrosome amplification inhibits paracrine invasion. Shown is the representative image of MDA‐MB468 cells that crossed the matrigel‐coated transwell upon co‐culturing with control or sgANGPTL4 MCF10A cells with centrosome amplification. Scale bars, 50 μm. (g, h) Centrosome amplification upregulates the expression of hypoxia and DMOG‐induced genes. GSEA revealed strong enrichment of an annotated hypoxia hallmark gene set (g) and DMOG‐induced genes (h) in cells with centrosome amplification. RNA‐Seq datasets being compared are indicated at the bottom of the GSEA plots for RPE‐1 cells. (i) The HIF‐1α monoclonal antibody is specific. Representative images of HIF‐1α in DMOG‐treated, DMSO (vehicle) and DMOG‐treated sgHIF‐1α cells. (j) HIF‐1α cytoplasmic fluorescence intensity is largely background staining. Automated quantification of cytoplasmic mean fluorescence intensity of HIF‐1α in DMOG‐treated, DMSO (vehicle) and DMOG‐treated sgHIF‐1α cells. For Cont DMSO, *n* = 210; Cont DMOG, *n* = 265; sgHIF‐1α DMOG, *n* = 464. (k) DMOG induces nuclear HIF‐1α. Automated quantification of nuclear HIF‐1α above cytoplasmic levels in DMOG‐treated, DMSO (vehicle) and DMOG‐treated sgHIF‐1α cells. For Cont DMSO, *n* = 210; Cont DMOG, *n* = 265; sgHIF‐1α DMOG, *n* = 464. (l) CRISPR‐mediated gene disruption of HIF‐1α does not affect the induction of extra centrosomes. The fraction of control (608) and PLK4 induced sgHIF‐1α MCF10A cells with more than two centrosomes, determined by immunostaining of centrin‐1. Data are means ± SEM from *n* = 2 independent experiments. (m) Induction of nuclear HIF‐1α after centrosome amplification‐induced senescence. Representative images of HIF‐1α in RPE‐1 cells with or without centrosome amplification. (n) CRISPR‐mediated gene disruption of HIF‐1α. HIF‐1α and GAPDH (loading control) immunoblots of lysates from doxycycline‐uninduced control PLK4, sg HIF‐1α PLK4, control 608 and sgHIF1A 608 cells which are DMOG‐treated. (o) The custom HIF‐1α signature up gene set is specific for centrosome amplification‐induced HIF‐1α activation. GSEA showing that CRISPR‐mediated gene disruption suppresses the custom HIF‐1α signature up gene set in sg HIF‐1α cells relative to control PLK4 MCF10A cells. (p) CRISPR‐mediated gene disruption of HIF‐1α in cells with centrosome amplification inhibits induction of paracrine invasion. Shown is the representative image of MDA‐MB468 cells that crossed the matrigel‐coated transwell upon co‐cultured with control or sg HIF‐1α MCF10A cells with centrosome amplification. Scale bars, 50 μm. All data are means ± SEM from three independent experiments analysed with one‐way ANOVA, Tukey’s multiple comparison test.
**Figure** **S3**. Centrosome amplification induced a SASP that constitutes HIF‐1α activation independent of prominent NF‐κB activity. (a‐e) Induction of senescence‐associated gene expression in cells with centrosome amplification cultured in physiological normoxia. By GSEA, RPE‐1 cells with centrosome amplification cultured in 5% O_2_ induce gene sets associated with the extracellular region (a), genes upregulated (b) and downregulated (c) in senescence, hypoxia (d) and the custom HIF‐1α signature gene set (e). (f, g) Centrosome amplification induces nuclear HIF‐1α in RPE‐1 cells when cells are cultured in 5% O_2_. Representative images (f) and quantification (g) of nuclear HIF‐1α in the indicated RPE‐1 cells. (h) Fold induction of ANGPTL4 and p21 from RNA‐Seq of cells cultured in 5% O_2_ relative to controls. (i) Lack of NF‐κB nuclear accumulation after centrosome amplification. Representative images of p65 RELA (NF‐κB) and DAPI (grey) in RPE‐1 with and without centrosome amplification as compared to a TNFα treatment as positive control.
**Figure** **S4**. A pathway linking centrosome amplification to HIF‐1α activation (a) Small molecule Rac inhibition prevents the accumulation of nuclear HIF‐1α after centrosome amplification. Representative images of the indicated MCF10A cells were treated with 50 μm NSC23766 or vehicle and HIF‐1α nuclear accumulation was measured. (b) CRISPR‐mediated gene disruption of TRIO. Trio and GAPDH (loading control) immunoblots of lysates from doxycycline‐uninduced control PLK4, sgTrio PLK4, control 608 and sgTrio 608 cells. (c) CRISPR‐mediated gene disruption of Trio does not affect the induction of extra centrosomes. The fraction of control 608 and PLK4 induced sgTRIO MCF10A cells with more than two centrosomes, determined by immunostaining of centrin‐1. Data are means ± SEM from *n* = 2 independent experiments. (d) siRNA knockdown of p22^phox^. p22^phox^ and GAPDH (loading control) immunoblots of lysates from doxycycline‐uninduced control PLK4, p22^phox^ knockdown PLK4, control 608 and p22^phox^ knockdown 608 cells. (e) siRNA knockdown of p22^phox^ does not affect the induction of extra centrosomes. The fraction of control (608) and PLK4‐induced p22^phox^ knockdown MCF10A cells with more than 2 centrosomes, determined by immunostaining of centrin‐1. Data are means ± SEM from *n* = 2 independent experiments. (f) siRNA knockdown of p22^phox^ prevents the accumulation of nuclear HIF‐1α after centrosome amplification. Representative images of HIF‐1α of the indicated MCF10A cells. (g) TRIO is required for the upregulation of hypoxia‐induced genes in MCF10A cells with centrosome amplification. Shown is a GSEA plot comparing cells with centrosome amplification with or without TRIO gene disruption. (h) CRISPR‐mediated gene disruption of Trio does not significantly affect the regulation of HIF‐1α and hypoxia‐induced genes in MCF10A control 608 cells. Shown are the normalised enrichment score and false discovery rate q‐values of HIF‐1α and hypoxia‐induced genes for sgTrio 608 cells relative to control 608 cells. Scale bars, 50 μm.
**Figure** **S5**. The role of hydrogen peroxide in the activation of HIF‐1α after centrosome amplification and activation of HIFα by gamma irradiation and oncogene‐induced senescence. (a) Accumulation of superoxide after centrosome amplification. Representative images of dihydroethidium labelling of superoxide levels in MCF10A cells with and without centrosome amplification. Pyocyanin treatment is the positive control. (b) Conversion of superoxide into hydrogen peroxide further induces nuclear HIF1α accumulation in cells with centrosome amplification. Representative images of HIF‐1α in the indicated MCF10A cells after treatment with TEMPOL. (c) GSEA showing that catalase treatment prevents the upregulation of the hallmark hypoxia gene set in MCF10A cells. (d, e) Nuclear HIF‐1α levels are significantly increased in senescent cells 8 days post‐γ‐irradiation. Representative images (d) and quantification (e) of nuclear HIF‐1α (grey) in control and senescent MCF10A cells. (f‐i) GSEA showing that the hallmark hypoxia gene sets and custom HIFα signature up (HIF‐1α) are upregulated in MCF10A cells 8 days post‐γ‐irradiation (f, g) and IMR90 cells undergoing oncogene‐induced senescence (Acosta et al., 2013) (h, i) Scale bars, 50 μm. Data are means ± SEM from *n* = 3 independent experiments, *p < 0.05; analysed with Student’s t test.

**Table S1:**Supporting information.

## Data Availability

The data that support the findings of this study are openly available in Annotare at https://www.ebi.ac.uk/arrayexpress/experiments/E‐MTAB‐11126, reference number [E‐MTAB‐11126].

## APPENDIX A

METHODS

Cell culture All source cell culture was maintained in media containing plasmocin (1:5000 dilution, Invivogen) to prevent mycoplasma contamination. Culture plates were briefly coated with 40 μg/ml of fibronectin (Sigma), and glass coverslips were incubated in 40 μg/ml fibronectin for at least 2 h. Human mammary epithelial MCF10A cells were maintained at 37°C with 5% CO_2_ atmosphere and cultured as previously described (Godinho et al., 2014). In brief, MCF10A cells were cultured in phenol red‐free DMEM/F12 (Invitrogen) supplemented with 5% horse serum (Sigma), 20 ng/ml epidermal growth factor (EGF; Sigma), 10 mg/ml insulin (Invitrogen), 100 mg/ml hydrocortisone (Sigma), 1 ng/ml cholera toxin (Sigma) and 100 IU/ml penicillin and 100 μg/ml streptomycin (Invitrogen). Telomerase‐immortalised RPE‐1 cells (ATCC), and all derivative cell lines generated in this study were cultured in phenol red‐free DMEM/F12 media containing 10% FBS, 100 U/ml penicillin and 100 μg/ml streptomycin. RPE‐1 cells were maintained at 37°C in 5% CO_2_ atmosphere. For experiments performed in physiological normoxia, RPE‐1 cells were maintained at 5% O_2_ conditions in a custom‐designed hypoxia chamber (Coy Labs).

To conditionally overexpress PLK4, lentiviral vectors pLenti‐CMV‐TetR‐Blast (17,492, Addgene) and pLenti‐CMV/TO‐Neo‐Dest (17,292, Addgene) were used. Wild‐type PLK4 and PLK4 1–608 cDNAs (control 608) were cloned using the Gateway system into the pLenti‐CMV/TO‐Neo‐Dest vector Cells were coinfected with a lentivirus containing the TetR and with the lentivirus containing the wild‐type PLK4 or control 608 open reading frames. Transduced cells were selected with Blasticidin (10 μg/ml) and Geneticin (200 μg/ml). control 608 and PLK4 overexpression was induced transiently. Transduced cells that survive the selection are used for subsequent experiments and a maintenance dose of 5 μg/ml of Blasticidin and 80 μg/ml of Geneticin was used in stock cultures.

Generation of tetraploids and “evolved” tetraploids with normal centrosome numbers To generate G1 arrested tetraploids, 15 cm dishes were seeded with 6 million exponentially growing RPE1‐FUCCI cells, such that they were ∼65% confluent the following day. Day 2: 4 μM Dihydrocytochalasin B (DCB) was added to each 15 cm dish for 16 hr. Day 3: DCB‐treated cells were washed five times for 5 min, incubated in medium containing 2.5 μg/ml Hoechst dye for 1 hr and then trypsinised in 0.05% trypsin. G1 diploids (2C DNA content; mCherry+, GFP−) and G1 tetraploids (4C DNA content; mCherry+, GFP−) were isolated by FACS sorting. FACS‐sorted cells were seeded on 6‐well plates coated with 40 μg/ml fibronectin.

Evolved tetraploid RPE‐1 cells, kindly provided by Huadi Zhang, with the normal number of centrosomes were derived as described (Ganem et al., 2009). Briefly, tetraploid RPE‐1 cells were treated for 16 h with 0.2 μm cytochalasin D, and FACS‐sorted by DNA content using Hoechst staining (Molecular Probes). The peak of cells with a DNA content of 8C was isolated and cultured for ∼1 week before a second iteration of the FACS sorting to re‐isolate 8C cells. A total of five purifying cell sortings for ∼6 weeks were required to generate a pure population of proliferating tetraploid cells with normal centrosome numbers.

Induction of centrosome amplification and senescence At Day 0, control 608 and PLK4 cells were gently dissociated into single cells, then seeded at low density into media with 2 μg/ml of doxycycline such that they were ~ 80% confluent on Day 5. After ~48 h of doxycycline incubation, the culture media with doxycycline was replaced. On Day 5, cells are gently dissociated into single cells before reseeding into 6‐well plates to ~80% confluent on Day 7. Because control 608 cells grow faster than PLK4‐induced cells, control 608 cells are seeded at ~2.5 times lower density than PLK4‐induced cells. Cells at ~80% confluency are analysed either at Day 7 or Day 8. Media is left unchanged for ~3 days before analysis.

Antibodies, siRNA and drugs Primary antibodies used in this study were: mouse monoclonal antibodies against Centrin (1:500 for immunofluorescence; Merck Millipore catalogue number 04–1624, clone number 20H5), mouse monoclonal antibodies against ANGPTL4 (1:50 for Western blotting; Santa Cruz catalogue number sc‐373762, clone number C‐7), rabbit polyclonal antibodies against Trio, was a gift from A. Debant (University of Montpellier, France; 1:500 for western blotting), mouse monoclonal antibodies against Rb (Thermo Fisher Scientific catalogue number: BDB554136, clone number G3‐245), rabbit polyclonal antibodies against phosphoRb Ser780 (catalogue number 9307 S), mouse monoclonal antibodies against GAPDH (1:10000 for Western blotting; Abcam catalogue number ab8245), rabbit polyclonal antibodies against GAPDH (1:10000 for Western blotting; Abcam catalogue number ab9485), mouse monoclonal antibodies HIF‐1α (1:100 for Western blotting; BD Transduction Laboratories catalogue number 610959, clone number 54) rabbit polyclonal or mouse monoclonal antibodies against HIF‐1α (1:50–1:100 for immunofluorescence; Genetex catalogue number GTX127309, Abcam catalogue number ab16066), rabbit polyclonal antibodies against phosphoH2AX (1:500 for immunofluorescence; EMD Millipore Upstate catalogue number 05‐636‐I, Clone: JBW301), rabbit polyclonal antibodies against p65/RELA (1:500 for immunofluorescence; Santa Cruz Biotechnology catalogue number sc‐372), mouse monoclonal antibodies against CYBA (1:100 for Western blotting; Santa Cruz catalogue number sc‐271968), Mouse IgG antibody (Abcam, ab37355) was used at 40 μg/ml as control for blocking experiments. The ANGPTL4 mouse antibody mAb11F6C4, used at 40 μg/ml for blocking experiments, was a gift from Tan Nguan Soon, Andrew (Lee Kong Chian School of Medicine, Nanyang Technological University, Singapore). siRNA against CYBA p22phox (Santa Cruz), smartpool siRNA against LATS2 and p53 (Dharmacon).

Doxycycline (Sigma Aldrich catalogue number D3072) was used at 2 mg/ml. Catalase (Sigma Aldrich (catalogue number: C9322)) was used at 2 U/μl for 24 h. TNFα (Roche Diagnostics catalogue number: 11371843001) was used at 20 ng/ml for 2 h. The following doses of inhibitors were used: 50 μM NSC23766 (EMD Millipore), 4 μM of dihydrocytochalasin B (DCB; Sigma) and 40 ng/ml Doxorubicin (Sigma).

Immunofluorescence and microscopy Cells were fixed at 4°C with 4% paraformaldehyde in cytoskeleton stabilisation buffer (10 mM PIPES at pH 6.8, 100 mM KCl, 300 mM sucrose, 2 mM EGTA and 2 mM MgCl_2_) on ice for 25 min and subsequently permeabilised with 0.2% Triton‐X in PBS for 10 min at room temperature. Fixed cells were blocked with 5% BSA in TBS overnight at 4°C. Primary antibodies were incubated overnight at 4°C for HIF‐1α and 45 min room temperature for phosphoH2AX and p65/RELA. For centrin‐1 staining, cells were fixed with −20°C methanol for 5 min on ice. For all immunostaining, secondary Alexa Fluor antibodies were incubated at room temperature for an hour.

Images were collected with Nikon Ti inverted microscope with 20× wide‐field optics, a Hamamatsu ORCA ER cooled CCD camera controlled with Nikon NIS‐Element software and an OkoLab 37°C, 5% CO_2_ microscope chamber or with a 40× objective on the Nikon W1 Spinning Disk confocal microscope.

Matrigel‐coated transwell invasion assay A previously described assay for matrigel‐coated transwell invasion assay was adapted for the current study as follows. Eight micrometres Boyden chamber inserts were used (Cell Biolabs: CytoSelect Tumor Transwell ECM Migration Assay, Catalogue No. CBA‐216). For the bottom chamber, MCF10A cells with or without centrosome amplification that were cultured for 48 h in 400 μl of full media was used. mCherry MDA‐MB‐468 cells were seeded on the top chamber. After overnight culture, cells on top of each insert were removed and the transwell membrane was mounted onto slides. Migration of mCherry MDA‐MB‐468 cells to the bottom of the membrane was visualised with a spinning disc microscope at 10× magnification.

Paracrine senescence assay was performed as previously described (Acosta et al., 2013). In brief, cells were co‐cultured separated by transwells. After a week MCF10A wild‐type cells in the bottom were split, seeded and cultured alone for 14 additional days.

CRISPR Knockouts and Lentiviral Transduction Puromycin selectable CRISPR lentiviruses were obtained commercially from Applied Biological Materials. Three different CRISPR lentiviruses were tested for each target gene. The CRISPR lentivirus that achieves the best knockout and the MCF10A knockout cells were characterised and used for subsequent experiments. The guide RNA targeting the sequences of the genes of interest and scrambled control is as follow. Scrambled control catalogue number K011, Trio Target Sequence catalogue number K2470115, ANGPTL4 Target Sequence 3 catalogue number K0085515. For the above CRISPR constructs, polyclonal population of cells that were selected with puromycin were used for experiments.

The previously described (Cho et al., 2016) HIF‐1α pLenti‐CRISPRv2 was a gift from William G. Kaelin, Jr. (Dana‐Farber Cancer Institute, Boston, USA). The sgRNA sequence targeting HIF‐1α is as follow: ‘5’‐ CACCGTGTGAGTTCGCATCTTGATA ‐’3′. Clonal selection for single‐cell clones after puromycin selection were used for the experiments.

For lentivirus production, 6 μg lentiviral constructs, 3 μg psPAX2 (Addgene) and 3 μg pMD2.G (Addgene) were transfected into 293 T cells at 80% confluency in a 10‐cm dish with 8 ml of media. Twenty microlitre of lipofectamine 3000 (Life Technologies) was used, and transfection was performed according to the manufacturer's instructions. Virus was harvested at 48 h and 72 h post‐infection, and filtered with a 0.2 μm filter. MCF10A cells were infected for 12 h, with virus containing the genes of interest in the presence of 10 μg/ml polybrene, washed thoroughly and allowed to recover for 24 h before any selection.

Image processing and analysis ImageJ macros are created to determine the nuclear to cytoplasmic ratio of fluorescence intensity and the number of phosphoH2AX foci in cells. For the quantification of nuclear and cytoplasmic mean fluorescence intensity of HIF‐1α and p65, a nuclei mask was generated from the Z‐projection of the Hoescht nuclear staining and a 1 μm region surrounding the nucleus was designated as the cytoplasmic region. The watershed function was applied on the nuclei mask to ensure separation of overlapping nuclei.

For the quantification of γ‐H2AX foci, a rolling‐ball (5 pixels) background subtraction was applied to 8‐bit images labelled for γ‐H2AX. A nuclear mask generated from the Z‐projection of the Hoescht nuclear staining after watershed segmentation was used to identify the γ‐H2AX foci within an individual nucleus. The “Find Maxima” function on ImageJ was used to count the number of γ‐H2AX foci in each nucleus. The noise for the γ‐H2AX staining was obtained from cytoplasmic staining and set as the noise tolerance input on the “Find Maxima” function.

Suspended Cell Size Measurement, SA‐β‐gal assays and Superoxide Assay Suspended cell size was determined using the coulter counter (Beckman, Vi‐cell). SA‐β‐gal activity was determined using the Senescent Cell Staining Kit (Sigma), by staining SA‐β‐gal for overnight, according to the manufacturer's instructions. Cells positive for SA‐β‐gal were identified by visual scoring. Superoxide levels were measured using a superoxide assay (Abcam catalogue number: ab139476) according to manufacturer instructions.

NextSeq500 RNA Library Preparation and Sequencing RNA quantity was determined on the Qubit using the Qubit RNA Assay Kit (Life Tech), and RNA quality was determined on the Bioanalyzer using the RNA Pico Kit (Agilent). Using the NEB Next Ultra RNA Library Prep Kit for Illumina (NEB), which selects for poly (A) mRNA, 100 ng of total RNA was used for cDNA library construction, according to the manufacturer's protocol. Following library construction, DNA quantity was determined using the Qubit High Sensitivity DNA Kit (Life Technologies) and library insert size was determined using the Bioanalyzer High Sensitivity Chip Kit (Agilent). Finally, qPCR was performed using the Universal Library Quantification Kit for Illumina (Kapa Biosystems) and ran on the 7900HT Fast qPCR machine (ABI) for quantification and normalisation of libraries. Libraries that passed quality control were diluted to 2 nM DNA content using sterile water and then sequenced on the NextSeq500 (Illumina) at a final concentration of 12pM, according to the manufacturer's instructions.

RNA Sequencing Analysis Sequencing reads were aligned to the reference genome using the RNA‐specific STAR aligner using default parameters (Dobin et al., 2013). The quality of the sequencing run and alignment was assessed by RNA‐SeQC (DeLuca et al., 2012). The featureCounts tool is used to count sequencing reads mapping to the reference genome at the gene level (Liao et al., 2014). The counts are subsequently normalised between samples using DESeq2 to control for experimental variation (Anders & Huber, 2010). Differential expression analysis was performed between the desired experimental contrasts using DESeq2.

Ingenuity Pathway Analysis Meta‐analysis of the DESeq2 analysed RNA sequencing data was performed to identify the top pathways that are statistically associated with the list of the significantly induced genes (Papathanasiou et al., 2015) (fold change ≥2, FDR q‐value <0.05, using the Ingenuity Pathway Analysis platform IPA, Ingenuity Systems, Qiagen).

Gene Set Enrichment Analysis We used GSEA v3.0 to examine the association between gene sets and gene expression. Normalised read counts from DESeq2 analysis were used for GSEA. Genes with a 0 read count in either control or experimental group were excluded. GSEA was performed using gene sets as permutation type, 1000 permutations and log2 ratio of classes as metrics for ranking genes. We employ the hallmark gene sets from the Molecular Signatures Database (MSigDB) (Subramanian et al., 2005; Liberzon et al., 2015; Liberzon et al., 2011). Hallmark gene sets summarise and represent specific well‐defined biological states or processes and display coherent expression. These gene sets were generated by a computational methodology based on identifying gene set overlaps and retaining genes that display coordinate expression, thus reducing data noise and redundancy.

To construct a HIF‐1α signature up gene set, we performed RNA sequencing of MCF10A cells after overnight treatment with 1 μm of DMOG, with DMSO‐treated cells as control. We included genes that were upregulated by DMOG in DESeq analysis (*p*(adjusted) < 0.05, fold change ≥4, Table S1).

Quantitative RT‐PCR Total RNA was isolated using the RNAeasy Mini Kit (Qiagen), and cDNA was synthesised using SuperScript III (Invitrogen) according to manufacturer instructions. Quantitative RT‐qPCR was performed in triplicate using SYBR Green (Life Technologies) on a ViiA 7 Real‐Time PCR machine (Thermo Fisher Scientific) with GAPDH as an internal normalisation control.

qPCR primers (PrimeTime^®^ Predesigned qPCR Assays) purchased from IDT were used and product information were as follow: GAPDH, Assay ID: Hs.PT.39a.22214836; Angptl4, Assay ID: Mm.PT.58.12348309.

ELISA Cells were starved for 2 h before reintroducing full media to collect cellular secretion. After 24 h of incubation, the conditioned (full) media is collected and a protease inhibitor cocktail (Sigma) was added to prevent protein degradation. The conditioned media was centrifuged for 10 min at 5000 rpm to remove any cellular debris and subsequently quantified for ANGPTL4's concentration with ELISA (ELH‐ANGPTL4, RayBiotech). The total cell number was used to normalise ANGPTL4's concentration across replicates.
