# Erratum to: The variant senescence‐associated secretory phenotype induced by centrosome amplification constitutes a pathway that activates hypoxia‐inducible factor‐1α

**DOI:** 10.1111/acel.13991

**Published:** 2023-09-11

Wu, S. K., Ariffin, J., Chian, T. S., & Picone, R. (2023). The variant senescence‐associated secretory phenotype induced by centrosome amplification constitutes a pathway that activates hypoxia‐inducible factor‐1α. *Aging Cell*, 22, e13766. https://doi.org/10.1111/acel.13766.

In the published version of Wu et al (2023), the current affiliation, Mechanobiology Institute & Department of Biological Sciences, National University of Singapore, Singapore is incorrectly linked to the authors' Juliana Arrifin and Remigio Picone instead of Selwin K. Wu.

The present address should be displayed as follows:

**Present address.**

Selwin K. Wu, Mechanobiology Institute & Department of Biological Sciences, National University of Singapore, Singapore.

