# Correction to “The Variant Senescence‐Associated Secretory Phenotype Induced by Centrosome Amplification Constitutes a Pathway That Activates Hypoxia‐Inducible Factor‐1α”

**DOI:** 10.1111/acel.70567

**Published:** 2026-05-27

Wu, S. K., Ariffin, J., Chian, T. S., & Picone, R. (2023). The Variant Senescence‐Associated Secretory Phenotype Induced by Centrosome Amplification Constitutes a Pathway That Activates Hypoxia‐Inducible Factor‐1α. *Aging Cell*, 22, e13766. https://doi.org/10.1111/acel.13766.

In Figure 4a, the representative image corresponding to the 608 mutant under the p53 knockdown condition was inadvertently used for both the control and p53 knockdown panels due to mislabeling during figure assembly. The correct image for the control condition is provided here. This error does not affect other data in the manuscript or the conclusions of the study.

We apologize for this error.

**FIGURE 4:**
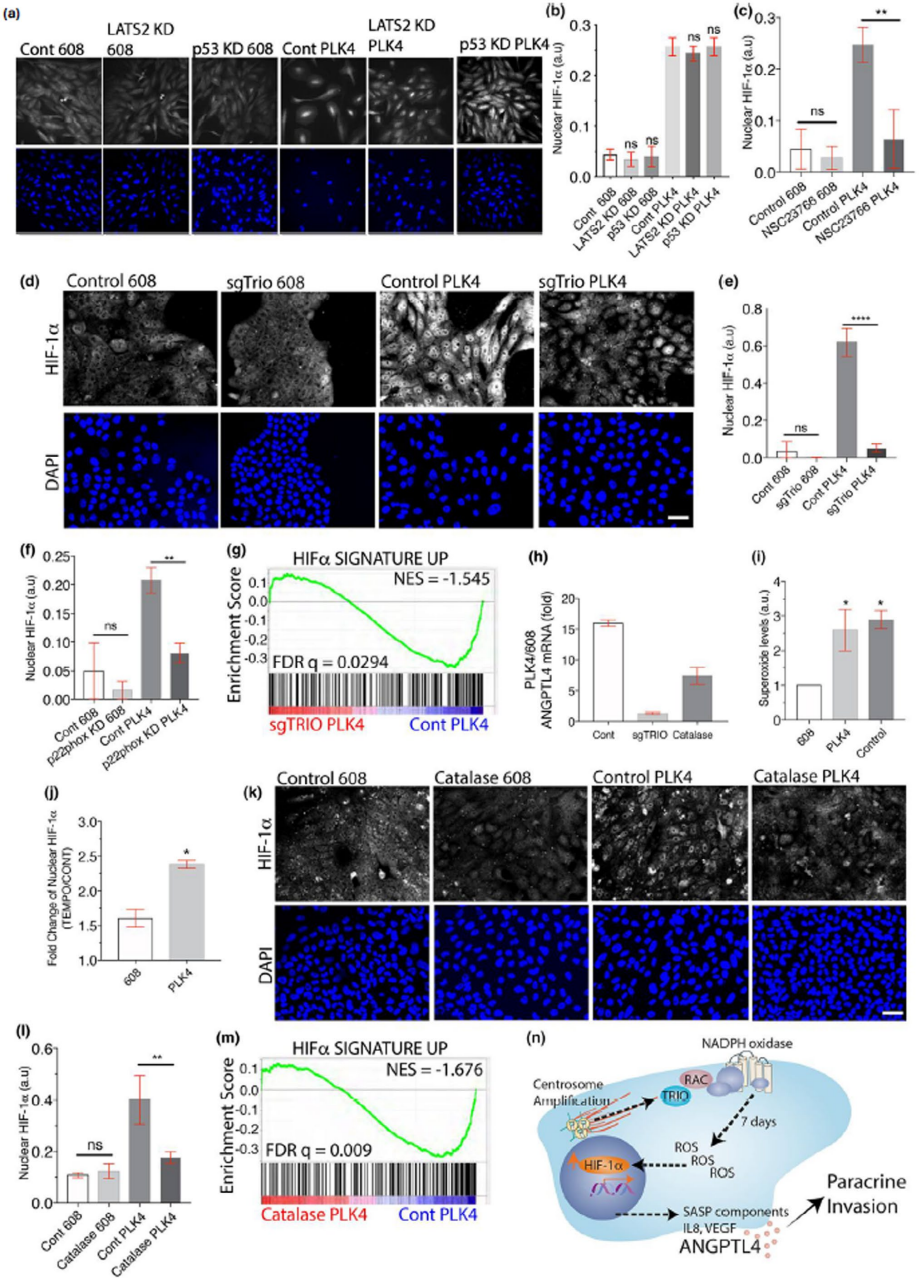
Centrosome amplification‐induced ROS. (a, b) RNAi‐mediated knockdown of p53 or LATS2 does not affect nuclear HIF‐1α accumulation after centrosome amplification. Shown are representative images (a) and quantification (b) of nuclear HIF‐1α levels in the indicated cells. (c) Small molecule Rac‐1 inhibition prevents the accumulation of nuclear HIF‐1α after centrosome amplification. The indicated MCF10A cells were treated with 50 μm NSC23766 or vehicle and HIF‐1α nuclear accumulation was measured. (d, e) CRISPR‐mediated gene disruption of TRIO blocks nuclear HIF‐1α accumulation after centrosome amplification. Gene targeting of a pool of cells was performed in the indicated MCF10A cells prior to the initiation of centrosome amplification. Shown are representative image (d) and quantification (e) of nuclear HIF‐1α levels in the indicated cells. (f) siRNA knockdown of p22phox inhibits nuclear HIF‐1α accumulation after centrosome amplification. (g) Trio is required for the upregulation of HIF‐1α‐induced genes in MCF10A cells with centrosome amplification. Shown is a GSEA plot comparing cells with centrosome amplification with or without TRIO gene disruption. (h) The induction of ANGPTL4 by centrosome amplification requires Trio and ROS. Shown is the fold induction of ANGPTL4 from RNA‐Seq after centrosome amplification in MCF10A cells after the indicated treatments. (i) Accumulation of superoxide after centrosome amplification. Superoxide levels were measured by dihydroethidium labelling in MCF10A cells with and without centrosome amplification. Pyocyanin treatment is the positive control. (j) Conversion of superoxide into hydrogen peroxide further induces nuclear HIF‐1α accumulation in cells with centrosome amplification. Shown are the fold changes in nuclear HIF‐1α in the indicated MCF10A cells after treatment with TEMPOL. (k, l) Catalase blocks the nuclear accumulation of HIF‐1α in cells with centrosome amplification. Representative images (k) and quantification (l) of HIF‐1α in the indicated MCF10A cells with and without catalase medium addition. (m) GSEA showing that catalase treatment prevents the upregulation of the custom HIF‐1α signature up gene set in MCF10A cells. All data are means ± SEM from *n* = 3 independent experiments, *p < 0.05, **p < 0.01, ***p < 0.001, ****p < 0.0001; analysed with one‐way ANOVA, Tukeys multiple comparison test. Scale bars, 50 μm. (n) Model for centrosome amplification‐induced SASP.

