# Supplementary material for: The variant senescence‐associated secretory phenotype induced by centrosome amplification constitutes a pathway that activates hypoxia‐inducible factor‐1α
Source: Aging Cell. 2023 Jan 20;22(3):e13766. doi: 10.1111/acel.13766 (PMC10014068; doi:10.1111/acel.13766)
Supplement: Supplementary file 1 — Figure S1 Features of the centrosome amplification SASP. (a, b) Fraction of control (608) and PLK4‐induced MCF10A (a) or RPE‐1 (b) cells with more than two centrosomes was determined by immunostaining of centrin‐1. Representative images and quantification of MCF10A cells with (PLK4) and without (608) centrosome amplification. Scale bars, 5 μm. Data are means ± SEM from n = 2 independent experiments. (c) Centrosome amplification alters expression of genes related to cell motility and senescence. Ingenuity Pathway Analysis (IPA) of gene expression changes in RPE‐1 cells with centrosome amplification relative to controls revealing the top pathways altered by centrosome amplification. Hepatic Fibrosis/ Hepatic Stellate Cell Activation is a SASP‐regulated process (Krizhanovsky et al., 2008). (d, e) Induction of the expression of secreted proteins in cells with centrosome amplification. Heatmap showing the leading‐edge enrichment of the top 50 extracellular protein expression upregulated in MCF10A (d) and RPE‐1 (e) cells with centrosome amplification relative to control. (f, g) Induction of secreted protein expression in cells with centrosome amplification. Gene set enrichment analysis (GSEA) revealed enrichment of genes annotated to be the extracellular region in tetraploids relative to either parental diploids (f) or evolved tetraploids (g). NES: normalised enrichment score; FDR: false discovery rate. (h, i) Centrosome amplification alters expression of genes related to senescence. Ingenuity Pathway Analysis (IPA) of gene expression changes in tetraploids relative to either parental diploids (h) or evolved tetraploids (i) revealing the top pathways altered by centrosome amplification. *Hepatic Fibrosis/ Hepatic Stellate Cell Activation is a senescence‐regulated process (Krizhanovsky et al., 2008). (j, k) Heatmap showing the leading‐edge enrichment of the top 50 extracellular protein expression upregulated in tetraploids relative to either parental diploids (j) or evolve [file ACEL-22-e13766-s002.pdf]

Figure S1.

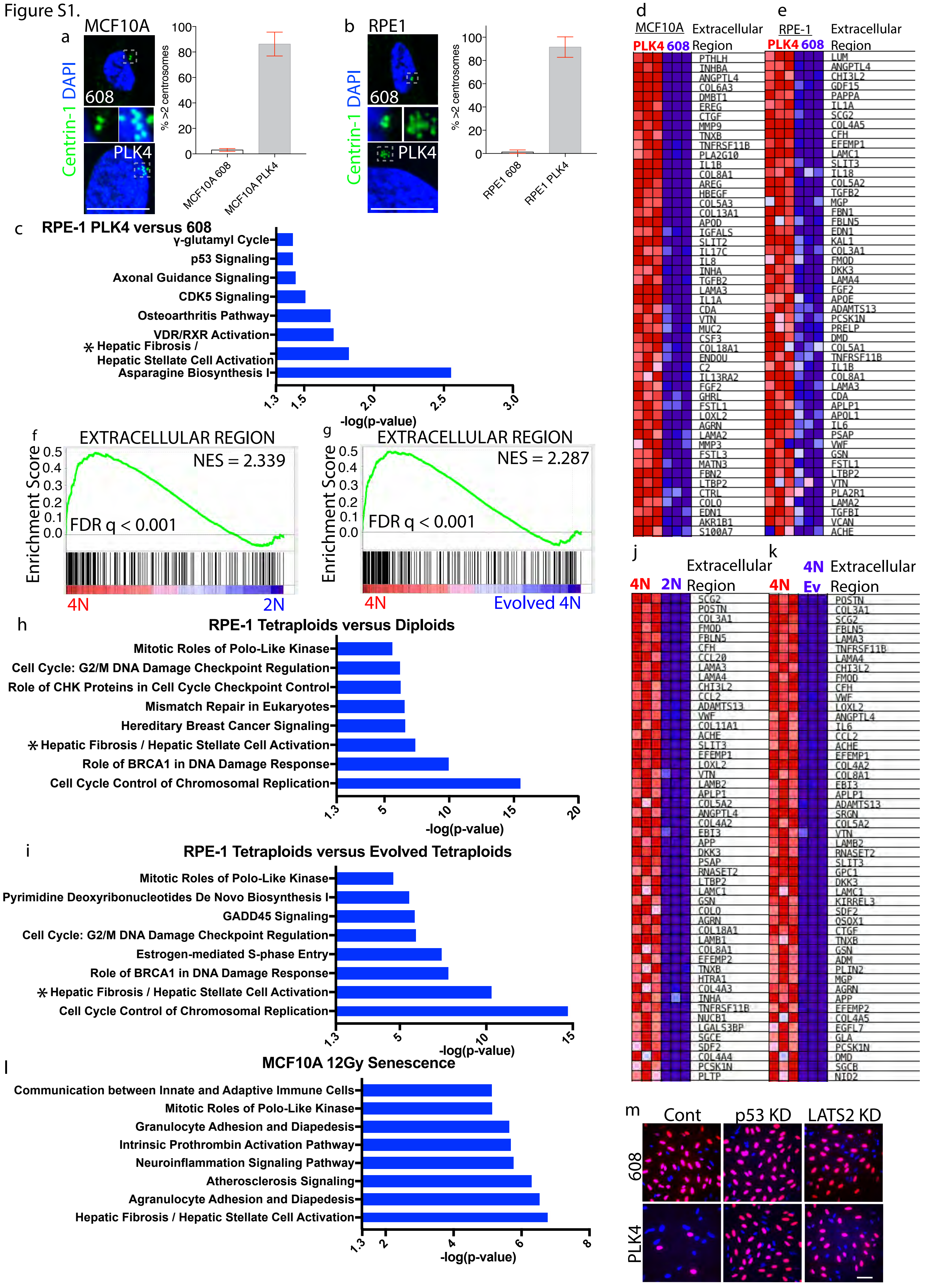

a
MCF10A
Centrin-1 DAPI
608
PLK4
% >2 centrosomes
MCF10A 608
MCF10A PLK4
b
RPE1
608
PLK4
RPE1 608
RPE1 PLK4
c
RPE-1 PLK4 versus 608
γ-glutamyl Cycle
p53 Signaling
Axonal Guidance Signaling
CDK5 Signaling
Osteoarthritis Pathway
VDR/RXR Activation
Hepatic Fibrosis / Hepatic Stellate Cell Activation
Asparagine Biosynthesis I
-log(p-value)
d
MCF10A Extracellular Region
PLK4 608
PTHLH INHBA ANGPTL4 COL6A3 DMBT1 EREG CTGF MMP9 TNXB TNFRSF11B PLA2G10 IL1B COL8A1 AREG HBEGF COL5A3 COL13A1 APOD IGFALS SLIT2 IL17C IL8 INHA TGFB2 LAMA3 IL1A CDA VTN MUC2 CSF3 COL18A1 ENDOU C2 IL13RA2 FGF2 GHRL FSTL1 LOXL2 AGRN LAMA2 MMP3 FSTL3 MATN3 FBN2 LTBP2 CTRL COLQ EDN1 AKR1B1 S100A7
e
RPE-1 Extracellular Region
PLK4 608
LUM ANGPTL4 CHI3L2 GDF15 PAPPA IL1A SCG2 COL4A5 CFH EFEMP1 LAMC1 SLIT3 IL18 COL5A2 TGFB2 MGP FBN1 FBLN5 EDN1 KAL1 COL3A1 FMOD DKK3 LAMA4 FGF2 APOE ADAMTS13 PCSK1N PRELP DMD COL5A1 TNFRSF11B IL1B COL8A1 LAMA3 CDA APLP1 APOL1 IL6 PSAP VWF GSN FSTL1 LTBP2 VTN PLA2R1 LAMA2 TGFBI VCAN ACHE
f
EXTRACELLULAR REGION
NES = 2.339
FDR q < 0.001
Enrichment Score
4N
2N
g
EXTRACELLULAR REGION
NES = 2.287
FDR q < 0.001
Enrichment Score
4N
Evolved 4N
h
RPE-1 Tetraploids versus Diploids
Mitotic Roles of Polo-Like Kinase
Cell Cycle: G2/M DNA Damage Checkpoint Regulation
Role of CHK Proteins in Cell Cycle Checkpoint Control
Mismatch Repair in Eukaryotes
Hereditary Breast Cancer Signaling
Hepatic Fibrosis / Hepatic Stellate Cell Activation
Role of BRCA1 in DNA Damage Response
Cell Cycle Control of Chromosomal Replication
-log(p-value)
i
RPE-1 Tetraploids versus Evolved Tetraploids
Mitotic Roles of Polo-Like Kinase
Pyrimidine Deoxyribonucleotides De Novo Biosynthesis I
GADD45 Signaling
Cell Cycle: G2/M DNA Damage Checkpoint Regulation
Estrogen-mediated S-phase Entry
Role of BRCA1 in DNA Damage Response
Hepatic Fibrosis / Hepatic Stellate Cell Activation
Cell Cycle Control of Chromosomal Replication
-log(p-value)
j
4N 2N Extracellular Region
SCG2 POSTN COL3A1 FMOD FBLN5 CFH CCL20 LAMA3 LAMA4 CHI3L2 CCL2 ADAMTS13 VWF COL11A1 ACHE SLIT3 EFEMP1 LOXL2 VTN LAMB2 APLP1 COL5A2 ANGPTL4 COL4A2 EBI3 APP DKK3 PSAP RNASET2 LTBP2 LAMC1 GSN COLQ AGRN COL18A1 LAMB1 COL8A1 EFEMP2 TNXB HTRA1 COL4A3 INHA TNFRSF11B NUCB1 LGALS3BP SGCE SDF2 COL4A4 PCSK1N PLTP
k
4N Ev Extracellular Region
POSTN COL3A1 SCG2 FBLN5 LAMA3 TNFRSF11B LAMA4 CHI3L2 FMOD CFH VWF LOXL2 ANGPTL4 IL6 CCL2 ACHE EFEMP1 COL4A2 COL8A1 EBI3 APLP1 ADAMTS13 SRGN COL5A2 VTN LAMB2 RNASET2 SLIT3 GPC1 DKK3 LAMC1 KIRREL3 SDF2 QSOX1 CTGF TNXB GSN ADM PLIN2 MGP AGRN APP EFEMP2 COL4A5 EGFL7 GLA PCSK1N DMD SGCB NID2
l
MCF10A 12Gy Senescence
Communication between Innate and Adaptive Immune Cells
Mitotic Roles of Polo-Like Kinase
Granulocyte Adhesion and Diapedesis
Intrinsic Prothrombin Activation Pathway
Neuroinflammation Signaling Pathway
Atherosclerosis Signaling
Agranulocyte Adhesion and Diapedesis
Hepatic Fibrosis / Hepatic Stellate Cell Activation
-log(p-value)
m
Cont
p53 KD
LATS2 KD
608
PLK4

Figure S1 Features of the centrosome amplification SASP. (a, b) Fraction of control (608) and PLK4-induced MCF10A (a) or RPE-1 (b) cells with more than two centrosomes was determined by immunostaining of centrin-1. Representative images and quantification of MCF10A cells with (PLK4) and without (608) centrosome amplification. Scale bars, 5 μm. Data are means ± SEM from n = 2 independent experiments. (c) Centrosome amplification alters expression of genes related to cell motility and senescence. Ingenuity Pathway Analysis (IPA) of gene expression changes in RPE-1 cells with centrosome amplification relative to controls revealing the top pathways altered by centrosome amplification. Hepatic Fibrosis/ Hepatic Stellate Cell Activation is a SASP-regulated process (Krizhanovsky et al., 2008). (d, e) Induction of the expression of secreted proteins in cells with centrosome amplification. Heatmap showing the leading-edge enrichment of the top 50 extracellular protein expression upregulated in MCF10A (d) and RPE-1 (e) cells with centrosome amplification relative to control. (f, g) Induction of secreted protein expression in cells with centrosome amplification. Gene set enrichment analysis (GSEA) revealed enrichment of genes annotated to be the extracellular region in tetraploids relative to either parental diploids (f) or evolved tetraploids (g). NES: normalised enrichment score; FDR: false discovery rate. (h, i) Centrosome amplification alters expression of genes related to senescence. Ingenuity Pathway Analysis (IPA) of gene expression changes in tetraploids relative to either parental diploids (h) or evolved tetraploids (i) revealing the top pathways altered by centrosome amplification. *Hepatic Fibrosis/ Hepatic Stellate Cell Activation is a senescence-regulated process (Krizhanovsky et al., 2008). (j, k) Heatmap showing the leading-edge enrichment of the top 50 extracellular protein expression upregulated in tetraploids relative to either parental diploids (j) or evolved tetraploids (k). (l) γ-irradiation alters expression of genes related to DNA damage-induced senescence. Ingenuity Pathway Analysis (IPA) of gene expression changes in MCF10A cells exposed to 12Gy of γ-irradiation relative to controls revealing the top pathways altered by DNA damage-induced senescence. (m) siRNA knockdown of p53 and LATS2 release MCF10A cells with centrosome amplification from proliferation arrest. Representative images of control, p53 knockdown and LATS2 knockdown MCF10A cells with (PLK4) and without (608) centrosome amplification that cycled through S-phase (24 hr. EdU-label, red). Scale bar, 50 μm.

Figure S2

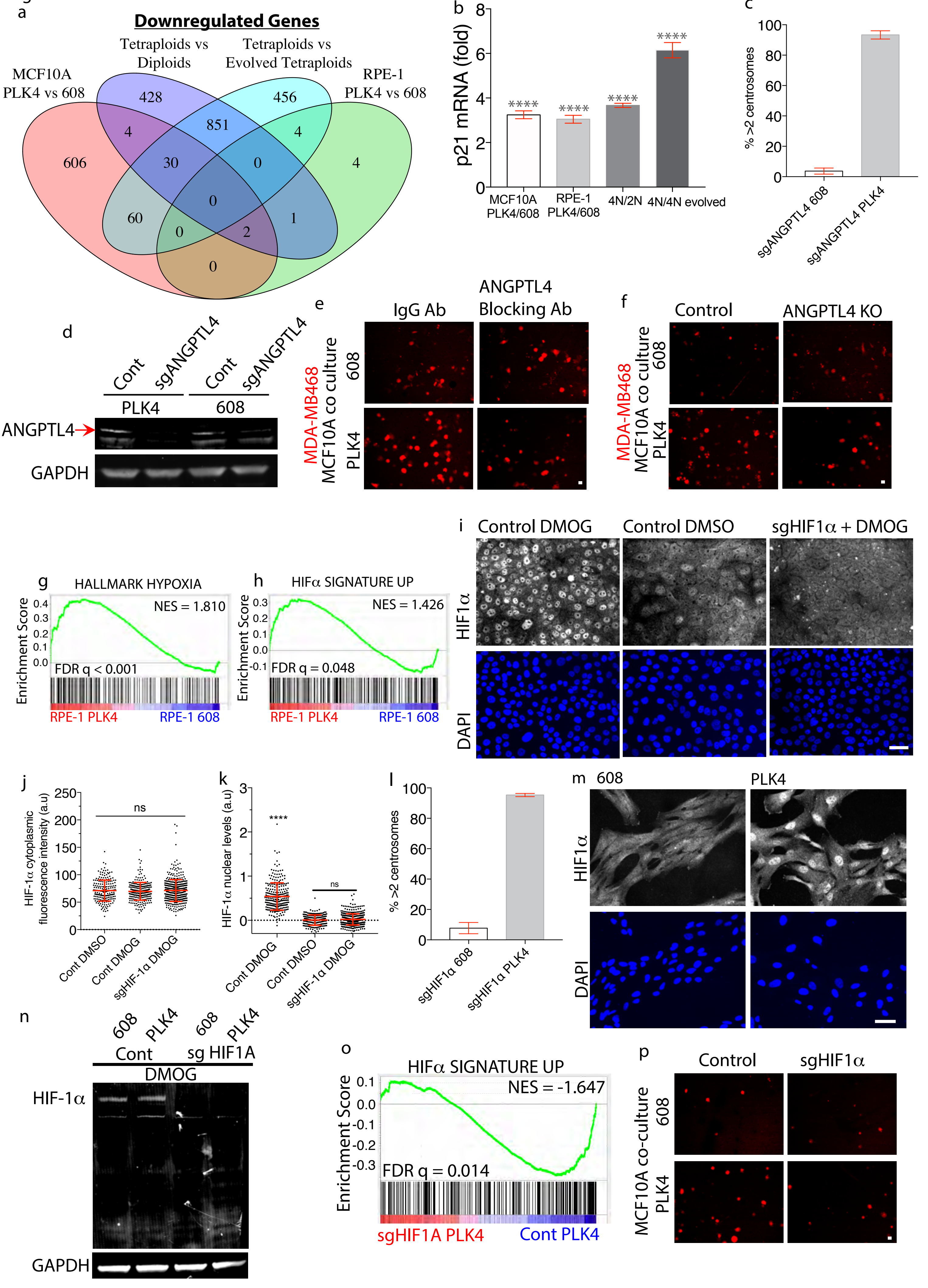

a
Downregulated Genes
Tetraploids vs Diploids
Tetraploids vs Evolved Tetraploids
MCF10A PLK4 vs 608
RPE-1 PLK4 vs 608
428
456
606
4
851
4
30
0
4
0
60
0
2
1
0
b
p21 mRNA (fold)
****
MCF10A PLK4/608
RPE-1 PLK4/608
4N/2N
4N/4N evolved
c
% >2 centrosomes
sgANGPTL4 608
sgANGPTL4 PLK4
d
Cont
sgANGPTL4
PLK4
608
ANGPTL4
GAPDH
e
IgG Ab
ANGPTL4 Blocking Ab
MDA-MB468
MCF10A co culture
608
PLK4
f
Control
ANGPTL4 KO
g
HALLMARK HYPOXIA
NES = 1.810
FDR q < 0.001
Enrichment Score
RPE-1 PLK4
RPE-1 608
h
HIFα SIGNATURE UP
NES = 1.426
FDR q = 0.048
i
Control DMOG
Control DMSO
sgHIF1α + DMOG
HIF1α
DAPI
j
HIF-1α cytoplasmic fluorescence intensity (a.u)
ns
Cont DMSO
Cont DMOG
sgHIF-1α DMOG
k
HIF-1α nuclear levels (a.u)
****
ns
l
% >2 centrosomes
sgHIF1α 608
sgHIF1α PLK4
m
608
PLK4
n
608
PLK4
Cont
sg HIF1A
DMOG
HIF-1α
GAPDH
o
HIFα SIGNATURE UP
NES = -1.647
FDR q = 0.014
sgHIF1A PLK4
Cont PLK4
p
Control
sgHIF1α
MCF10A co-culture

Figure S2. Gene expression altered by centrosome amplification, a functional requirement for ANGPTL4 (a) Zero genes are commonly downregulated by centrosome amplification from all experimental conditions. Venn diagram for the indicated comparisons showing the overlap of downregulated genes from RNA-Seq (twofold change, $q < 0.05$). (b) Induction of p21 expression in cells with centrosome amplification. p21 mRNA fold changes from RNA-Seq in RPE-1 and MCF10A cells in PLK4 versus 608, tetraploids versus parental diploids and tetraploids versus evolved tetraploids. Data are means ± SEM from n = 3 independent experiments, ****$p < 0{:}0001$; adjusted p-value (grey) analysed with DESeq2 Wald test. (c) CRISPR-mediated gene disruption of ANGPTL4 does not affect the induction of extra centrosomes. The fraction of control (608) and PLK4 induced sgANGPTL4 MCF10A cells with more than two centrosomes, determined by immunostaining of centrin-1. Data are means ± SEM from n = 2 independent experiments. (d) CRISPR-mediated gene disruption of ANGPTL4. ANGPTL4 and GAPDH (loading control) immunoblots of lysates from doxycycline-uninduced control PLK4, sgANGPTL4 PLK4, control 608 and sgANGPTL4 608 cells. (e) Inhibition of ANGPTL4 compromises paracrine invasion stimulated by cells with centrosome amplification. Shown is the representative image of MDA-MB468 cells in the bottom chamber of the transwell in the presence of either control IgG or an ANGPTL4 blocking antibody, co-cultured with the indicated MCF10A cells. (f) CRISPR-mediated gene disruption of ANGPTL4 in cells with centrosome amplification inhibits paracrine invasion. Shown is the representative image of MDA-MB468 cells that crossed the matrigel-coated transwell upon co-culturing with control or sgANGPTL4 MCF10A cells with centrosome amplification. Scale bars, 50 μm. (g, h) Centrosome amplification upregulates the expression of hypoxia and DMOG-induced genes. GSEA revealed strong enrichment of an annotated hypoxia hallmark gene set (g) and DMOG-induced genes (h) in cells with centrosome amplification. RNA-Seq datasets being compared are indicated at the bottom of the GSEA plots for RPE-1 cells. (i) The HIF-1α monoclonal antibody is specific. Representative images of HIF-1α in DMOG-treated, DMSO (vehicle) and DMOG-treated sgHIF-1α cells. (j) HIF-1α cytoplasmic fluorescence intensity is largely background staining. Automated quantification of cytoplasmic mean fluorescence intensity of HIF-1α in DMOG-treated, DMSO (vehicle) and DMOG-treated sgHIF-1α cells. For Cont DMSO, n = 210; Cont DMOG, n = 265; sgHIF-1α DMOG, n = 464. (k) DMOG induces nuclear HIF-1α. Automated quantification of nuclear HIF-1α above cytoplasmic levels in DMOG-treated, DMSO (vehicle) and DMOG-treated sgHIF-1α cells. For Cont DMSO, n = 210; Cont DMOG, n = 265; sgHIF-1α DMOG, n = 464. (l) CRISPR-mediated gene disruption of HIF-1α does not affect the induction of extra centrosomes. The fraction of control (608) and PLK4 induced sgHIF-1α MCF10A cells with more than two centrosomes, determined by immunostaining of centrin-1. Data are means ± SEM from n = 2 independent experiments. (m) Induction of nuclear HIF-1α after centrosome amplification-induced senescence. Representative images of HIF-1α in RPE-1 cells with or without centrosome amplification. (n) CRISPR-mediated gene disruption of HIF-1α. HIF-1α and GAPDH (loading control) immunoblots of lysates from doxycycline-uninduced control PLK4, sg HIF-1α PLK4, control 608 and sgHIF1A 608 cells which are DMOG-treated. (o) The custom HIF-1α signature up gene set is specific for centrosome amplification-induced HIF-1α activation. GSEA showing that CRISPR-mediated gene disruption suppresses the custom HIF-1α signature up gene set in sg HIF-1α cells relative to control PLK4 MCF10A cells. (p) CRISPR-mediated gene disruption of HIF-1α in cells with centrosome amplification inhibits induction of paracrine invasion. Shown is the representative image of MDA-MB468 cells that crossed the matrigel-coated transwell upon co-cultured with control or sg HIF-1α MCF10A cells with centrosome amplification. Scale bars, 50 μm. All data are means ± SEM from three independent experiments analysed with one-way ANOVA, Tukey's multiple comparison test.

Figure S3

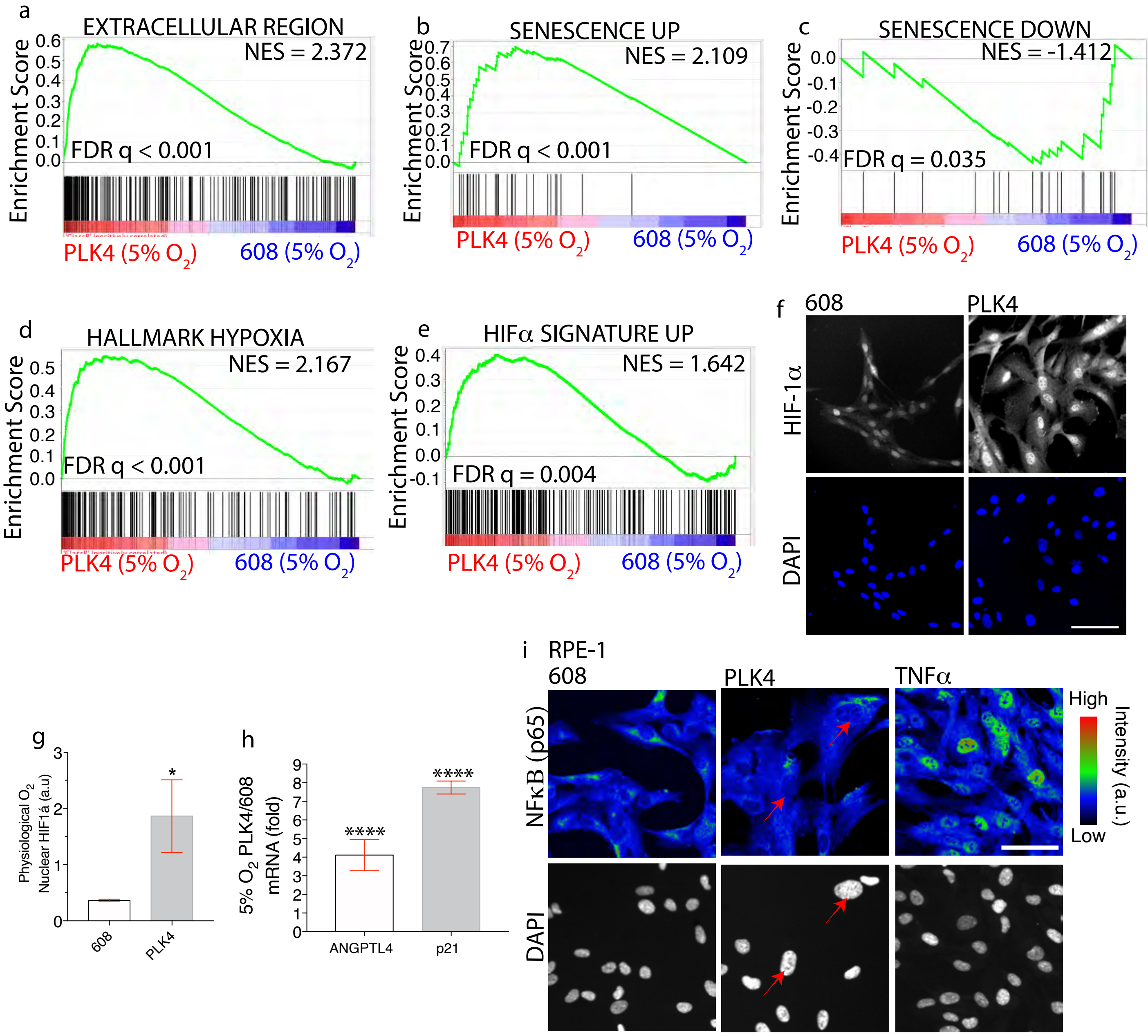

Figure S3. Centrosome amplification induced a SASP that constitutes HIF-1α activation independent of prominent NF-κB activity. (a-e) Induction of senescence-associated gene expression in cells with centrosome amplification cultured in physiological normoxia. By GSEA, RPE-1 cells with centrosome amplification cultured in 5% O2 induce gene sets associated with the extracellular region (a), genes upregulated (b) and downregulated (c) in senescence, hypoxia (d) and the custom HIF-1α signature gene set (e). (f, g) Centrosome amplification induces nuclear HIF-1α in RPE-1 cells when cells are cultured in 5% O2. Representative images (f) and quantification (g) of nuclear HIF-1α in the indicated RPE-1 cells. (h) Fold induction of ANGPTL4 and p21 from RNA-Seq of cells cultured in 5% O2 relative to controls. (i) Lack of NF-κB nuclear accumulation after centrosome amplification. Representative images of p65 RELA (NF-κB) and DAPI (grey) in RPE-1 with and without centrosome amplification as compared to a TNFα treatment as positive control.

Figure S4

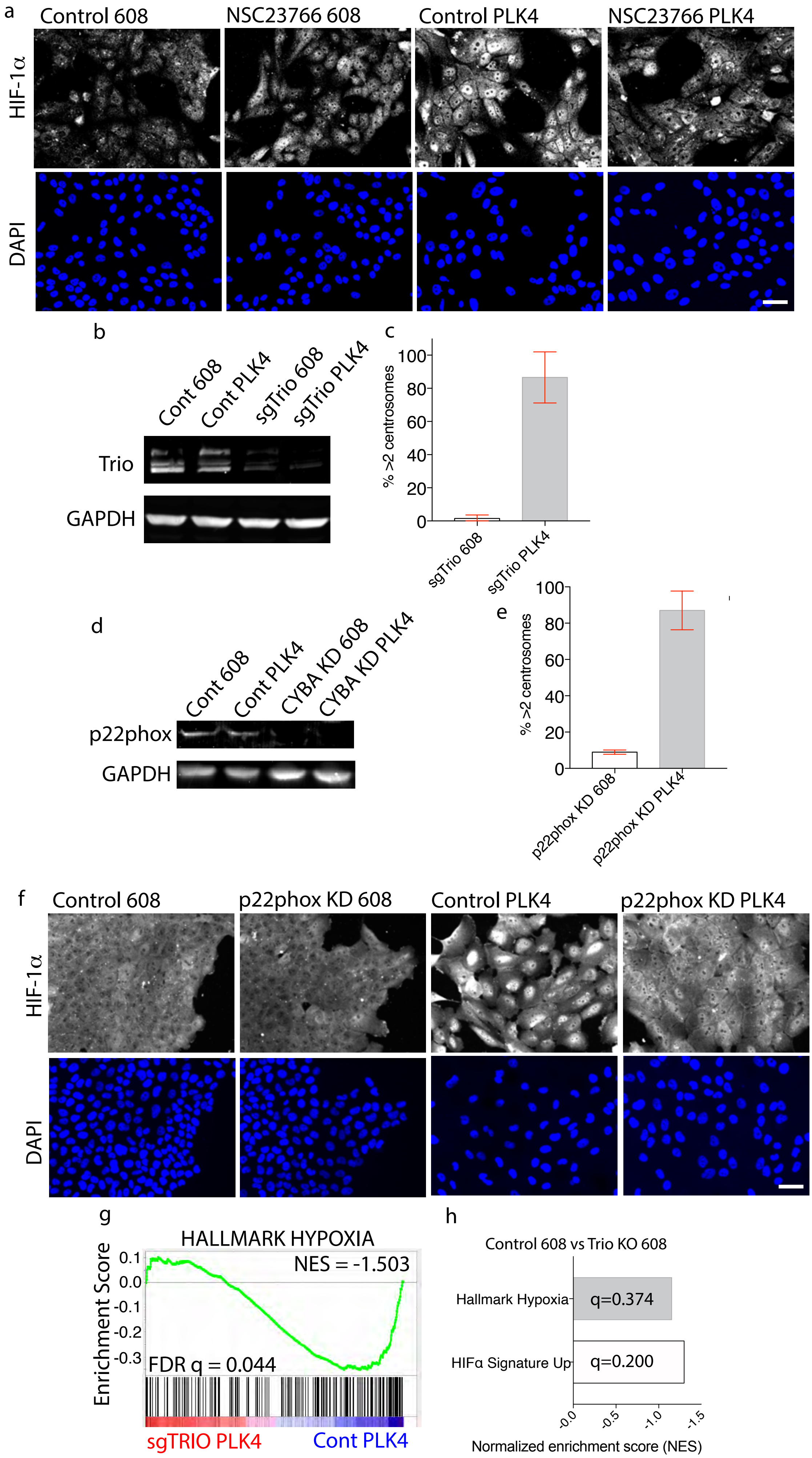
a
Control 608
NSC23766 608
Control PLK4
NSC23766 PLK4
HIF-1α
DAPI
b
Cont 608
Cont PLK4
sgTrio 608
sgTrio PLK4
Trio
GAPDH
c
% >2 centrosomes
0
20
40
60
80
100
sgTrio 608
sgTrio PLK4
d
Cont 608
Cont PLK4
CYBA KD 608
CYBA KD PLK4
p22phox
GAPDH
e
% >2 centrosomes
0
20
40
60
80
100
p22phox KD 608
p22phox KD PLK4
f
Control 608
p22phox KD 608
Control PLK4
p22phox KD PLK4
HIF-1α
DAPI
g
HALLMARK HYPOXIA
Enrichment Score
0.1
0.0
-0.1
-0.2
-0.3
NES = -1.503
FDR q = 0.044
sgTRIO PLK4
Cont PLK4
h
Control 608 vs Trio KO 608
Hallmark Hypoxia
q=0.374
HIFα Signature Up
q=0.200
-0.0
-0.5
-1.0
-1.5
Normalized enrichment score (NES)

Figure S4. A pathway linking centrosome amplification to HIF-1α activation (a) Small molecule Rac inhibition prevents the accumulation of nuclear HIF-1α after centrosome amplification. Representative images of the indicated MCF10A cells were treated with 50 μm NSC23766 or vehicle and HIF-1α nuclear accumulation was measured. (b) CRISPR-mediated gene disruption of TRIO. Trio and GAPDH (loading control) immunoblots of lysates from doxycycline-uninduced control PLK4, sgTrio PLK4, control 608 and sgTrio 608 cells. (c) CRISPR-mediated gene disruption of Trio does not affect the induction of extra centrosomes. The fraction of control 608 and PLK4 induced sgTRIO MCF10A cells with more than two centrosomes, determined by immunostaining of centrin-1. Data are means ± SEM from n = 2 independent experiments. (d) siRNA knockdown of p22phox. p22phox and GAPDH (loading control) immunoblots of lysates from doxycycline-uninduced control PLK4, p22phox knockdown PLK4, control 608 and p22phox knockdown 608 cells. (e) siRNA knockdown of p22phox does not affect the induction of extra centrosomes. The fraction of control (608) and PLK4-induced p22phox knockdown MCF10A cells with more than 2 centrosomes, determined by immunostaining of centrin-1. Data are means ± SEM from n = 2 independent experiments. (f) siRNA knockdown of p22phox prevents the accumulation of nuclear HIF-1α after centrosome amplification. Representative images of HIF-1α of the indicated MCF10A cells. (g) TRIO is required for the upregulation of hypoxia-induced genes in MCF10A cells with centrosome amplification. Shown is a GSEA plot comparing cells with centrosome amplification with or without TRIO gene disruption. (h) CRISPR-mediated gene disruption of Trio does not significantly affect the regulation of HIF-1α and hypoxia-induced genes in MCF10A control 608 cells. Shown are the normalised enrichment score and false discovery rate q-values of HIF-1α and hypoxia-induced genes for sgTrio 608 cells relative to control 608 cells. Scale bars, 50 μm.

Figure S5

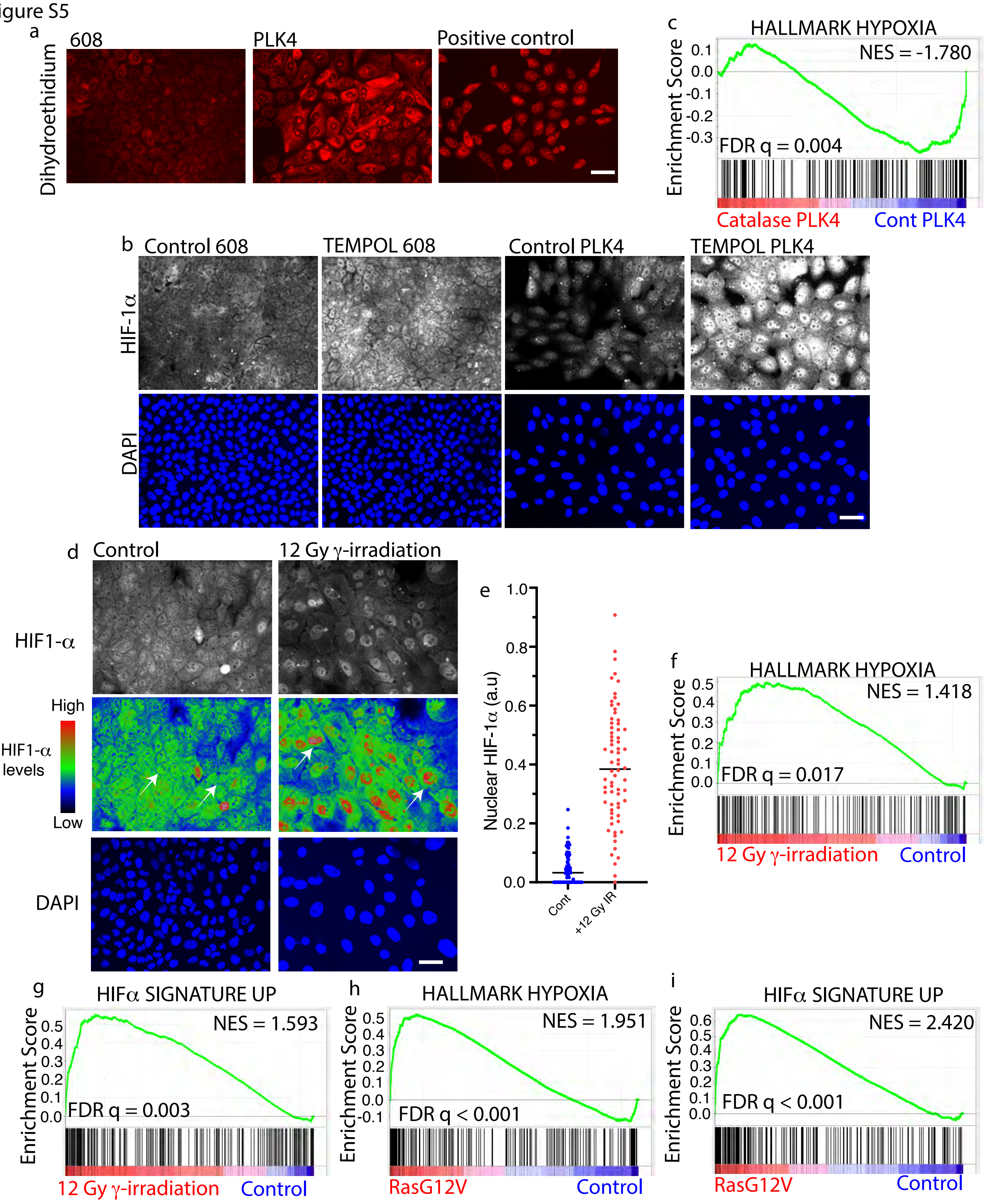
a
608
PLK4
Positive control
Dihydroethidium
b
Control 608
TEMPOL 608
Control PLK4
TEMPOL PLK4
HIF-1α
DAPI
c
HALLMARK HYPOXIA
NES = -1.780
FDR q = 0.004
Enrichment Score
Catalase PLK4
Cont PLK4
d
Control
12 Gy γ-irradiation
HIF1-α
High
HIF1-α levels
Low
DAPI
e
Nuclear HIF-1α (a.u)
Cont
+12 Gy IR
f
HALLMARK HYPOXIA
NES = 1.418
FDR q = 0.017
Enrichment Score
12 Gy γ-irradiation
Control
g
HIFα SIGNATURE UP
NES = 1.593
FDR q = 0.003
Enrichment Score
12 Gy γ-irradiation
Control
h
HALLMARK HYPOXIA
NES = 1.951
FDR q < 0.001
Enrichment Score
RasG12V
Control
i
HIFα SIGNATURE UP
NES = 2.420
FDR q < 0.001
Enrichment Score
RasG12V
Control

Figure S5. The role of hydrogen peroxide in the activation of HIF-1α after centrosome amplification and activation of HIFα by gamma irradiation and oncogene-induced senescence. (a) Accumulation of superoxide after centrosome amplification. Representative images of dihydroethidium labelling of superoxide levels in MCF10A cells with and without centrosome amplification. Pyocyanin treatment is the positive control. (b) Conversion of superoxide into hydrogen peroxide further induces nuclear HIF1α accumulation in cells with centrosome amplification. Representative images of HIF-1α in the indicated MCF10A cells after treatment with TEMPOL. (c) GSEA showing that catalase treatment prevents the upregulation of the hallmark hypoxia gene set in MCF10A cells. (d, e) Nuclear HIF-1α levels are significantly increased in senescent cells 8 days post-γ-irradiation. Representative images (d) and quantification (e) of nuclear HIF-1α (grey) in control and senescent MCF10A cells. (f-i) GSEA showing that the hallmark hypoxia gene sets and custom HIFα signature up (HIF-1α) are upregulated in MCF10A cells 8 days post-γ-irradiation (f, g) and IMR90 cells undergoing oncogene-induced senescence (Acosta et al., 2013) (h, i) Scale bars, 50 μm. Data are means ± SEM from n = 3 independent experiments, $^*p < 0.05$; analysed with Student's t test.
